# Changes in the Rumen Bacteriome Structure and Enzymatic Activities of Goats in Response to Dietary Supplementation with *Schizochytrium* spp.

**DOI:** 10.3390/microorganisms9071528

**Published:** 2021-07-17

**Authors:** Alexandros Mavrommatis, Dimitrios Skliros, Emmanouil Flemetakis, Eleni Tsiplakou

**Affiliations:** 1 Laboratory of Nutritional Physiology and Feeding, Department of Animal Science, School of Animal Biosciences, Agricultural University of Athens, GR-11855 Athens, Greece; mavrommatis@aua.gr; 2 Laboratory of Molecular Biology, Department of Biotechnology, School of Food, Biotechnology and Development, Agricultural University of Athens, GR-11855 Athens, Greece; dsklhros@gmail.com (D.S.); mflem@aua.gr (E.F.)

**Keywords:** goat, rumen, microbiome, 16S rRNA, microalgae, amylase, protease, cellulase, Illumina

## Abstract

With the aim to produce functional dairy products enriched with polyunsaturated fatty acids (PUFA) by using feed supplements, radical changes could occur in the rumen microbiome. This work investigated the alterations of the rumen bacteriome of goats fed with PUFA-rich marine microalgae *Schizochytrium* spp. For the trial, twenty-four goats were divided into four homogenous clusters (six goats/treatment) according to their fat-corrected (4%) milk yield, body weight, and age; they were individually fed with alfalfa hay and a concentrate (F/C = 50/50). The concentrate of the control group (CON) contained no microalgae, while those of the treated groups were supplemented daily with 20 (ALG20), 40 (ALG40), and 60 g (ALG60) of *Schizochytrium* spp./goat. Rumen fluid samples were collected using a stomach tube during the 20th and 40th days of the experiment. The microbiome analysis using a 16S rRNA sequencing platform revealed that Firmicutes were decreased in microalgae-fed goats, while Bacteroidetes showed a tendency to increase in the ALG40 group due to the enhancement of *Prevotellaceae*. Cellulolytic bacteria, namely *Treponema bryantii*, *Ruminococcus gauvreauii*, *R. albus*, and *R. flavefaciens*, were decreased in the ALG40 group, resulting in an overall decrease of cellulase activity. In contrast, the amylolytic potential was significantly enhanced due to an upsurge in *Ruminobacter amylophilus*, *Succinivibrio dextrinosolvens*, and *Fretibacterium fastidiosum* populations. In conclusion, supplementing goats’ diets with 20 g *Schizochytrium* spp. could be considered a sustainable and efficient nutritional strategy to modulate rumen microbiome towards the development of dairy products enriched with bioactive compounds, while higher levels induced substantial shifts in determinant microbes’ populations.

## 1. Introduction

The livestock sector aiming to follow the global markets and consumer trends regarding functional foods has focused on several bioactive compounds [1]. Amongst such bioactive compounds, microalgae are considered to be a novel and sustainable nutritional alternative capable of enriching ruminants’ milk with polyunsaturated fatty acids (PUFA) that are associated with consumers’ health benefits [2,3,4]. Notably, the dietary supplementation with *Schizochytrium* spp. enriched cow [5], sheep [6], and goat milk [7] with docosapentaenoic (DPA) and docosahexaenoic (DHA) fatty acids (FA). Interestingly, high-fat microalgae such as *Schizochytrium* spp. could also be used for their potential in reducing methane production in ruminants as reported in vitro by Fievez et al. [8]. Supporting this set of evidence, previous data signified the inhibiting potential of *Schizochytrium* spp. on methanogenic archaea adhered to feed particles [9] or floated in the liquid of the goats’ rumen [10].

Nevertheless, the reprogramming of the rumen microbiome by the inclusion of PUFA-rich feed supplements tends to holistically shift the core bacteriome, concealing significant risks towards rumen function and, consequently, animal performance. It has been observed that PUFAs of marine origin exert toxic effects on the cell membrane, particularly of Gram-positive bacteria [11], resulting in severe suppression of cellulolytic growth [12]. In this light, *Ruminococcus* species are considered to be the most sensitive taxa as they were unable to grow in the presence of DHA in in vitro cultures [12]. Thus, nutritional strategies for methane mitigation and biohydrogenation manipulation should also take into consideration the entire rumen bacteriome to ensure that fiber digestion is not considerably compromised. It is worth mentioning that the ruminants’ ability to degrade lignocellulose derived from fibrous feedstuffs appears to be of great importance classifying ruminants as the most sustainable livestock system considering the food–feed competition between humans and animals [13].

From this perspective, the multidisciplinary approaches that include but are not limited to the enrichment of milk and meat with bioactive molecules beneficial for human health, improved environmental sustainability through methane mitigation, and nitrogen utilization with the simultaneous guaranteed feed efficiency and animal performance shaping future livestock. However, multitargeted strategies require holistic tools, too. Until recently, the study of the rumen microbiome was implicitly linked with culture-based techniques, while most of the rumen microbes cannot be cultivated in pure cultures [14]. Therefore, these approaches are of little help when the goal is the elucidation of the relationships between community members [14]. The advent of meta-omics techniques such as 16S rRNA sequencing provides a much broader genomic and functional perspective in rumen microbial ecology. Up to now, the rumen bacteriome has been explored using metagenomic approaches (a) in vitro in cattle and sheep rumens in response to pure DPA, DHA, and eicosapentaenoic acid (EPA) fatty acids [15] and (b) in vivo in early life goats in response to dietary supplementation with microalgae *Schizochytrium* spp. [16]. However, limited information exists exploring the complicated interactions between rumen proteolytic, amylolytic, and cellulolytic bacteria.

Still, dependable discrimination of ruminal bacteria according to their degradation role appears to be unreliable in generating assumptions related to their utilization activity since the majority of them used to be involved in various procedures. For instance, *Butyrivibrio fibrisolvens* traditionally considered to have proteolytic, cellulolytic, and pectinolytic properties are also found to be important for PUFA biohydrogenation [12]. However, abundances of rumen microbes are not always correlated with their overall enzymatic potential or with their end products concentration. Specifically, *Fibrobacter succinogenes*, a primally cellulose-degrading bacterium, was found in a significantly lower abundance in the rumen of cows that were fed a high-concentrate ration, while carboxymethyl cellulose was prone to increase [17]. Furthermore, it has been observed that the composition rather than the absolute abundance of the methanogens in the rumen is firmly associated with CH_4_ production [18]. Thus, the bacteriome’s synergistic action on principal enzymatic activities related to feed fermentation should also be addressed for assessing the feasibility of such novel nutritional strategies in farm-scale conditions without compromising overall sustainability.

Even though the implementation of omics techniques in the rumen microbiome has launched a novel scientific field in animal science, there is still a need for advancing beyond the cataloging of rumen microbial populations and gaining a deeper understanding into the rumen microbial biochemical functions under the influence of certain dietary treatments [19]. Thus, this study aimed to evaluate the impact of dietary inclusion of *Schizochytrium* spp. at three different levels (20, 40, and 60 g/animal/day) on the key fermentation activities (amylolysis, proteolysis, and cellulolysis) by clustering rumen bacteriomes accordingly.

## 2. Materials and Methods

### 2.1. Experimental Design and Animals’ Diets

This study continued the analytical approach initiated in previous research works [7,9,10,20]. The experimental trial was conducted with respect to the guidelines of the European Union Directive on the protection of animals used for scientific purposes (EU 63/2010; Council of the European Union, 2010). Briefly, twenty-four 3–4-year-old dairy goats (local (Greek) × Alpine breeds) at 150 ± 10 days in milk (DIM) were grouped into four standardized subgroups (*n* = six goats/treatment) based on their fat-corrected milk (FCM6%) yield, age, and body weight (BW; 47.6 ± 5.9 kg). Each group was allocated to one of the following four groups: the control group (CON) receiving a basal diet consisting of 1 kg alfalfa hay and 1 kg concentrate/goat daily and the ALG20, ALG40, and ALG60 groups which were fed the same basal diet supplemented with 20 g, 40 g, and 60 g *Schizochytrium* spp./goat, respectively (Table 1). *Schizochytrium* spp. Is traded as a commercial product DHAgold (DSM Human Nutrition & Health, Heerlen, the Netherlands). Further information about ration design, microalgae inclusion, and chemical composition of the feeds was previously reported by Mavrommatis and Tsiplakou [7].

### 2.2. Rumen Samples Collection and DNA Extraction

Rumen samples were collected from five goats/treatment as previously described by Mavrommatis et al. [9] using an alternative to rumen cannulation, a nonsurgical and nonoperating procedure. Briefly, on days 20 and 40 of the trial, the rumen content was collected before morning feeding using a stomach tube (a flexible PVC tube with 1.5-mm thickness and 10-mm ID) and an electric vacuum pump (MZ2C, Vacuubrand Gmbh & Co Kg, Wertheim, Germany) as described for sheep and goats by Ramos Morales et al. [21]. Immediately after collection, pH of the rumen content was determined using a digital pH meter (pH 210 and a HI1236 electrode, Hanna Instruments, Smithfield, RI, USA) and the rumen digesta was filtered with cheesecloth layers to separate the liquid phase, and then the samples were frozen at −80 °C until further experimentation.

A total of 40 samples from 20 goats (five goats/treatment) at two sampling times were used (five out of six samples (goats) per treatment were randomly selected) for microorganisms’ DNA extraction as previously described by Mavrommatis et al. [9] using a modified cetyltrimethylammonium bromide (CTAB; Sigma-Aldrich, St. Louis, MO, USA) protocol. The aforementioned sampling points were selected based on the preliminary results of this study showing moderate microorganism adaptation after the 20th day [9].

### 2.3. PCR Amplification of the 16S rRNA Gene

Extensive quality assessment with a 2100 Bioanalyzer (Agilent Technologies, Santa Clara, CA, USA) and Nanodrop (Thermo Fisher Scientific, Waltham, MA, USA) was performed prior to libraries preparation. All DNA samples were of high quality and lacked PCR inhibitors and protein and RNA contamination. The 16S rRNA gene was targeted by PCR with primers (V3–V4) proposed by Klindworth et al. [22] (341F 5′-CCTACGGGNGGCWGCAG-3′ and 805R 5′-GACTACHVGGGTATCTAATCC-3). The samples were pooled and tagged for each library separately. PicoGreen™ (Quant-iT™ dsDNA Assay Kit, Thermo Fisher Scientific, Waltham, MA, USA) was used for each library for estimating DNA concentration with high accuracy (Table S1). The libraries were then inserted into a MiSeq platform (Illumina, San Diego, CA, USA) utilizing a 300 bp paired-end run method [23].

### 2.4. Bioinformatic Analysis

Paired-end (PE) data were merged with PEAR (v. 0.9.6) [24]. The primers used for PCR were trimmed with Cutadapt (v. 1.8.1) with default parameters [25]. Short sequences (<200 nts) were further trimmed from the data to avoid wrong taxonomical identification due to small contigs size. To ensure trimming of low-quality reads (threshold Q20), we used the Phred score and eliminated low-quality reads (Table S2). Furthermore, final reads were analyzed for chimera constructs due to the PCR procedure of Illumina sequencing using the UCHIME algorithm [26]. In order to reduce the complexity of the analysis, we annotated only the representative sequences. By using a threshold of 97% in nucleotide similarity and CD-HIT, we created a list of representative sequences, for which annotation was conducted [27]. For every sample, the sum of the taxonomical identifiers was assigned as operational taxonomic units (OTUs). In that way, OTUs distinguish bacteria at the genus level. Reads counts for each cluster were measured with the R package [28]. Each of the clean clustered sequences was compared against an rRNA database by using the BLASTn algorithm and the local alignment approach. The first hit for each cluster was considered as the best hit, and the annotation was completed. Clusters with fewer than seven sequences were treated as “no hit,” and the rest were named numerically. A rarefaction curve was obtained for each of the samples, and they were analyzed at the taxonomic level in order to identify if we reached the plateau of detection and were in a saturation situation when comparing the different samples (Figure S1).

### 2.5. Quantitative PCR Analysis for Validation

Validating the results obtained by DNA sequencing, representative bacteria species were also determined using a qPCR protocol. Changes in populations of *Butyrivibrio fibrisolvens*, *Fibrobacter succinogenes*, *Ruminobacter amylophilus*, and *Prevotella ruminicola* were calculated using a relative quantification method. The PCR platform, primer sets, amplifying regions, and quantification are available in the article by Mavrommatis et al. [9,10]. Figure S2 depicts the relative abundances of the above targets determined by qPCR and Illumina expressed as fold changes of the treated groups (ALG20, ALG40, and ALG60) compared to the control group (CON).

### 2.6. Rumen Enzymes and Ammonia Concentration

During rumen sample collection, 10 mL of the rumen digesta were filtered through four layers of cheesecloth and then centrifuged at 13,000× *g* at 4 °C for 5 min. The supernatant was stored in aliquots at −80 °C until the analysis. Each sample was defrosted only once to ensure enzyme functionality. The ammonia–nitrogen (NH_3_–N) concentration, alpha-amylase and protease activities were measured using a UV/Vis spectrophotometer (GENESYS 180, Thermo Fisher Scientific, Waltham, MA, USA). The NH_3_–N concentration was determined using a commercial BUN kit (BIOSIS, Athens, Greece) with proper calibrations using consecutive dilutions (10–100 mg/L) of a 20% ammonia solution (Thermo Fisher Scientific, Waltham, MA, USA) [29]. Alpha-amylase was assayed by monitoring the reduction of 3,5-dinitrosalicylic acid by released groups from starch at 540 nm according to the method of Worthington Biochemical Corporation. Protease activity was determined according to the method of Baintner [30]. Proteases split off colored azopeptides from azocasein. The residual azocasein, bacteria, etc. were precipitated with trichloroacetic acid (TCA; Sigma-Aldrich, St. Louis, MO, USA) and the red color of the azopeptides was then developed with alkali and measured at 440 nm. We attempted to evaluate cellulase activity using the well-described Azo-CM-Cellulose colorimetric method. However, it was impossible to generate reliable results. Hence, the Petri dish method described by Abe et al. [31] was used. Briefly, a medium containing 37 mM KH_2_PO_4_, 11 mM K_2_HPO_4_, 0.4 mM MgSO_4_·7H_2_O, 7.6 mM (NH_4_)_2_SO_4_, 27 mM microcrystalline cellulose at pH 5.5, and 15 g/L agar (*w*/*v*) was used. After the inoculation of the rumen fluid, the dishes were incubated at 50 °C for 16 h before evaluation. After this, 5 mL of the iodine solution were spread to visualize the hydrolytic halo. The same procedure was followed for xylanase activity based on the method of Kalim and Ali [32] with some modification. Briefly, the cellulase medium was used by substituting microcrystalline cellulose with 10 g/L xylan from corn core, while the incubation took place at 37 °C for 20 h. Standard curves were obtained by consecutive dilutions of endocellulase (*Aspergillus niger;* Megazyme Ltd., Wicklow, Ireland) and endo-1-4-beta-Xylanase M1 (*Trichoderma viride*; Megazyme Ltd., Wicklow, Ireland). The ImageJ densitometry software (version 1.6, National Institute of Health, Bethesda, MD, USA) was used for clearance zone quantitative analysis [33].

### 2.7. Statistical Evaluation of Data

**Figure 1:**
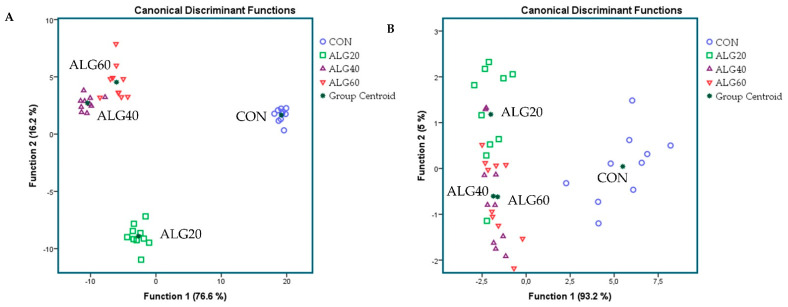
Discriminant plots separating (**A**) the four dietary treatments (CON; blue ○, ALG20; green □, ALG40; purple △, and ALG60; red ▽) according to pooled data of two sampling times (20th and 40th experimental day) that entered independents together on the abundances explained the forty-three most abundant genera in goats’ rumen (> 0.06%) and (**B**) discriminating the four dietary treatments (CON; blue ○, ALG20; green □, ALG40; purple △, and ALG60; red ▽) based on a step-wise method. CON = control concentrate without microalgae (*Schizochytrium* spp.); ALG20 = CON with 20 g/Kg *Schizochytrium* spp.; ALG40 = CON with 40 g/Kg *Schizochytrium* spp.; ALG60 = CON with 60 g/Kg *Schizochytrium* spp.

The dataset was analyzed using SPSS (v. 20.0; IBM). Discriminant analyses were performed to pool data of the first forty-three most abundant genera (>0.06%; highlighted with yellow in Table S5) to examine those variables capable of discriminating and ordering samples among (i) the four dietary groups (CON, ALG20, ALG40, and ALG60) as entered independents together (Figure 1A) and (ii) the four dietary groups based on a stepwise method (Figure 1B). Wilks’s lambda (λ) criterion was used for assessing discriminant functions. 

Dietary treatment effects on bacteriome abundances, diversity indices, ammonia concentration, and enzyme activities were investigated using a general linear model (GLM) for repeated measures (ANOVA) since the samples were collected at two sampling points (20th and 40th experimental days) from the same subjects (*n* = 40; 20 goats × two sampling points). Specifically, dietary groups (T = CON, ALG20, ALG40, and ALG60) were used as a fixed factor, sampling time (S)—as the repeated variable, while their interactions (T × S) were also included to evaluate the differences over time according to the following model:

Yijkl = µ + Ti + Sj + Ak + (T × S)ij + eijkl

where Yijk is the dependent variable, µ—the overall mean, Ti—the effect of dietary treatment (I = 4; CON, ALG20, ALG40, and ALG60), Sj—the effect of sampling time (j = 2; 20th and 40th experimental days), Ak—the animal’s random effect, (T × S)ij—the interaction between dietary treatments and sampling time, and eijk—the residual error. Post hoc analysis was performed when appropriate using a Tukey’s multiple range test. These results are extensively presented in the Supplementary Materials (Tables S3–S6 and S8).

**Figure 2:**
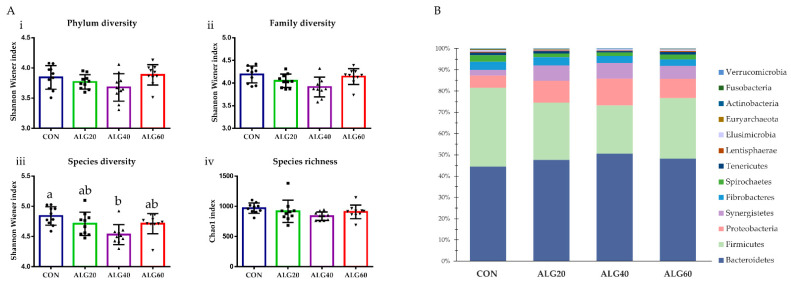
(**A**) Effects of supplementing with microalgae *Schizochytrium* spp. on (i) phylum, (ii) family, and (iii) species diversity based on the Shannon–Wiener index and (iv) Chao1 richness at the species level. Bars with different superscripts (a, b) mean dietary treatments differ significantly (*p* ≤ 0.05) according to the analysis of variance (ANOVA) using a general linear model for repeated measures; post hoc analysis was performed when appropriate using the Tukey’s multiple range test. (**B**) Relative abundances of the identified phyla (unidentified clusters and no-hits were omitted) in the four dietary treatments (CON, ALG20, ALG40, and ALG60) within the two sampling times (20th and 40th days) illustrated in the cumulative bar graph. After the exclusion of the unmapped clusters and no-hit sequences, the relative abundances of the identified phyla were transformed as the percentage of the total identified taxa. The mean relative abundances of the identified phyla, unmapped bacteria, and no-hit proportions are presented in Figure S3. CON = control concentrate without microalgae (*Schizochytrium* spp.); ALG20 = CON with 20 g/Kg *Schizochytrium* spp.; ALG40 = CON with 40 g/Kg *Schizochytrium* spp.; ALG60 = CON with 60 g/Kg *Schizochytrium* spp.

**Figure 3:**
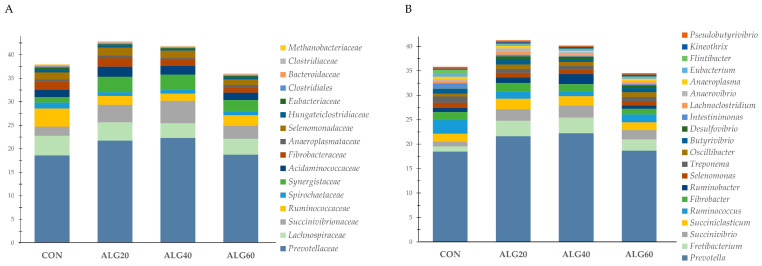
Relative abundances of the most dominant (**A**) families (>0.1%) and (**B**) genera (>0.3%) in the four dietary treatments (CON, ALG20, ALG40, and ALG60) within the two sampling times (20th and 40th days) illustrated in the cumulative bar graph. CON = control concentrate without microalgae (*Schizochytrium* spp.); ALG20 = CON with 20 g/Kg *Schizochytrium* spp.; ALG40 = CON with 40 g/Kg *Schizochytrium* spp.; ALG60 = CON with 60 g/Kg *Schizochytrium* spp.

**Figure 4:**
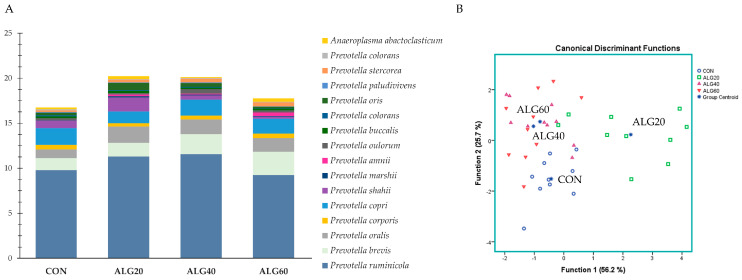
(**A**) Abundances of the most representative proteolytic species in the four dietary treatments (CON, ALG20, ALG40, and ALG60) within the two sampling times (20th and 40th days) illustrated in clustered stacked columns. (**B**) Discriminant plots separating the four dietary treatments (CON, blue ○; ALG20, green □; ALG40, purple △; and ALG60, red ▽) according to the pooled data of the two sampling times (20th and 40th experimental days) entered together as independents on the abundances of the sixteen principal species with proteolytic activity in the goats’ rumen. CON = control concentrate without microalgae (*Schizochytrium* spp.); ALG20 = CON with 20 g/Kg *Schizochytrium* spp.; ALG40 = CON with 40 g/Kg *Schizochytrium* spp.; ALG60 = CON with 60 g/Kg *Schizochytrium* spp.

**Figure 5:**
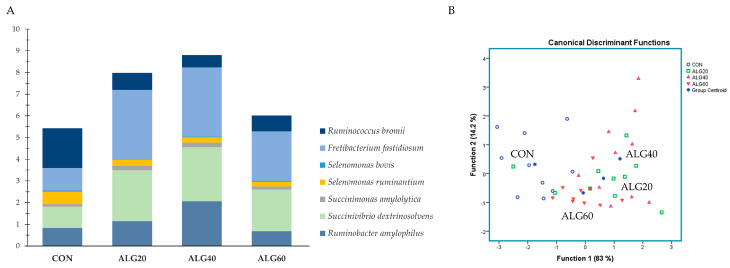
(**A**) Abundances of the most representative amylolytic species in the four dietary treatments (CON, ALG20, ALG40, and ALG60) within the two sampling times (20th and 40th days) illustrated in clustered stacked columns. (**B**) Discriminant plots separating the four dietary treatments (CON, blue ○; ALG20, green □; ALG40, purple △; and ALG60, red ▽) according to the pooled data of the two sampling times (20th and 40th experimental days) entered together as independents on the abundances of the seven principal species with amylolytic activity in the goats’ rumen. CON = control concentrate without microalgae (*Schizochytrium* spp.); ALG20 = CON with 20 g/Kg *Schizochytrium* spp.; ALG40 = CON with 40 g/Kg *Schizochytrium* spp.; ALG60 = CON with 60 g/Kg *Schizochytrium* spp.

**Figure 6:**
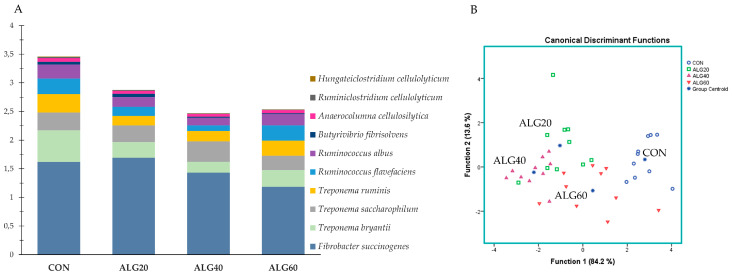
(**A**) Abundances of the most representative cellulolytic species in the four dietary treatments (CON, ALG20, ALG40, and ALG60) within the two sampling times (20th and 40th days) illustrated in clustered stacked columns. (**B**) Discriminant plots separating the four dietary treatments (CON, blue ○; ALG20, green □; ALG40, purple △; and ALG60, red ▽) according to the pooled data of the two sampling times (20th and 40th experimental days) entered together as independents on the abundances of the ten principal species with cellulolytic activity in the goats’ rumen. CON = control concentrate without microalgae (*Schizochytrium* spp.); ALG20 = CON with 20 g/Kg *Schizochytrium* spp.; ALG40 = CON with 40 g/Kg *Schizochytrium* spp.; ALG60 = CON with 60 g/Kg *Schizochytrium* spp.

**Figure 7:**
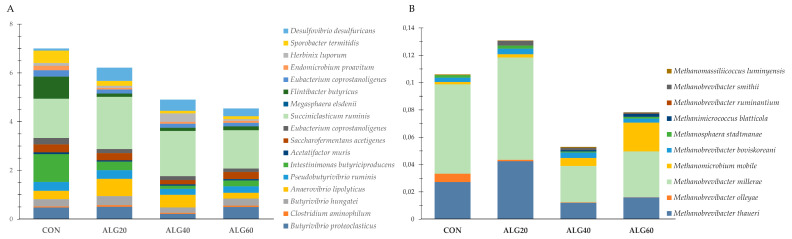
Abundances of (**A**) influential rumen species, (**B**) and methanogenic archaea in the four dietary treatments (CON, ALG20, ALG40, and ALG60) within the two sampling times (20th and 40th days) illustrated in clustered stacked columns. CON = control concentrate without microalgae (*Schizochytrium* spp.); ALG20 = CON with 20 g/Kg *Schizochytrium* spp.; ALG40 = CON with 40 g/Kg *Schizochytrium* spp.; ALG60 = CON with 60 g/Kg *Schizochytrium* spp.

GraphPad Prism 6.0 (2012) was used for interleaved bars for diversity and richness indices, enzyme activities, ammonia concentration, and ruminal pH, while errors bars represented the standard error of means (SEM), and the corresponding superscripts letters emerged from the SPSS repeated measures analysis (Figure 2A). Besides, aiming to further simplify the relative abundances of the bacteriome’s phyla, families, genera, and species, cumulative bar graphs were depicted using Microsoft Excel (Microsoft Office Professional Plus 2016). Specifically, the relative abundance of the rumen’s identified phyla are presented in Figure 2B as the percentage of the identified bacteria, omitting both unidentified taxa (unmapped) and no-hit sequences, while Figure S3 summarizes the identified, unidentified taxa and the sum of no-hit sequences. The proportion of the most abundant families (>0.1%) and genera (>0.3%) and the dominant species which were clustered according to their metabolic potential within the rumen (proteolytic, amylolytic, cellulolytic, methanogenic, etc.) are presented in their observed relative abundances (clustered stacked columns; Figures 3–7). Additionally, a Spearman’s correlation illustrated as a three-color heat map aimed to explain the relation between the dominant rumen genera and milk characteristics as previously reported by Mavrommatis and Tsiplakou [7].

## 3. Results

### 3.1. Dry Matter Intake

The dry matter intake of the concentrate was decreased significantly in the ALG60 goats compared to the other treatments (670 g vs. 1000 g) throughout the experimental period (Table S7). This led to a lower intake of microalgae than the scheduled one (40 vs. 60 g) since microalgae was added to the concentrate mixtures.

### 3.2. Sequencing, Quality Filtering, and Validation

A total of 2,638,775 reads was generated from the total of 40 samples, with the mean of 65,969 reads per sample. After quality filtering, 2,161,216 (82%) high-quality sequences remained, with the mean of 54,030 reads per sample. The average length was observed at 403 nts (±2.3 SD), while the mean quality was reported at 37.4 (±0.1 SD) (Table S2). Rarefaction curves of the bacterial population at the genus taxonomic level show that all the samples reached the plateau phase; thus, any further increase in the number of sequences would not impact the number of genera detected (Figure S1).

The dominant bacteria species featuring great importance in rumen functions were amplified using a well-established qPCR platform, validating the NGS results (Figure S2). The data in both cases were transformed as fold change compared to their control groups (Figure S2). Amongst the groups, the same pattern was observed between the qPCR and NGS data (Figure S2). Only for *Butyrivibrio fibrisolvens*, a minor discrepancy was found between qPCR and NGS in the ALG20 group; however, in both analyses, the differences were not significant (Figure S2).

### 3.3. Diversity, Richness, and Composition of the Ruminal Bacteriome

Figure 1A,B depicts the discriminant plots of the four dietary treatments (CON, blue ○; ALG20, green □; ALG40, purple △; and ALG60, red ▽) throughout the experimental period based on the forty-three most abundant genera (>0.06%) within the goats’ rumen. By inserting the variables independent-together, the proportions of the samples that were correctly classified were 100%, while Wilks’s λ was observed at < 0.001 for Function 1 (*p* < 0.001) and at 0.002 for Function 2 (*p* < 0.001) (Figure 1A). CON variables were significantly (Function 1) different from those of the microalgae-fed groups, while ALG20 was further allocated to the left bottom of the plot, indicating a dose response between 20 and 40 g of *Schizochytrium* spp. dietary supplementation of the rumen bacteriome (Figure 1A). Applying a stepwise method aiming to avoid any multicollinearity bias, a lower correct classification was achieved (80%). Wilks’s λ was observed at 0.042 for Function 1 (*p* < 0.001) and at 0.513 for Function 2 (*p* = 0.012), while the abundances of *Intestinimonas*, *Flintibacter*, *Anaerovibrio*, *Butyrivibrio*, *Anaerocolumna*, and *Endomicrobium* were the variables that contributed the most (Figure 1B). CON variables were significantly (Function 1) different from those of the microalgae-fed groups, while ALG20, ALG40, and ALG60 showed a minor overlap (Function 2), indicating that there was no significant dose-dependent effect considering the stepwise method (Figure 1B). Furthermore, discriminant plots based on the identified phyla and families are also available in Figure S4.

The Shannon–Wiener index did not differ between the phyla (*p* = 0.171), families (*p* = 0.055), and genera (*p* = 0.146), while in the ALG40 group, Shannon diversity at the species level (*p* = 0.033) was decreased significantly compared to the CON group (Figure 2A). Despite the significant reduction in species diversity, Chao1 richness in ALG40 was decreased only numerically (970 vs. 837; *p* = 0.271) (Figure 2A). The dominant phyla appeared to be Bacteroidetes (CON, 18.86%; ALG20, 21.91%; ALG40, 22.50%; ALG60, 18.92%), Firmicutes (CON, 15.66%; ALG20, 12.35%; ALG40, 10.07%; ALG60, 11.19%), and Proteobacteria, while some minor populations of Synergistetes, Fibrobacteres, Spirochaetes, Tenericutes, Lentisphaerae, Elusimicrobia, Euryarchaeota, Actinobacteria, Fusobacteria, and Verrucomicrobia were also identified (Figure 2B). Actinobacteria were significantly decreased (*p* = 0.021) in the ALG40 and ALG60-fed goats compared to the CON group (Table S3). Bacteroidetes, the predominant rumen phylum, tended to increase (*p* = 0.099) by 19% in the ALG40-fed goats compared to those that consumed the control diet (Table S3). Firmicutes were decreased (*p* = 0.012) in the microalgae-fed goats compared to the CON group (Table S3). Proteobacteria were increased significantly (*p* = 0.019) in the ALG20- and ALG40-fed goats compared to the CON group, while the clusters unable to be annotated were significantly lower (*p* < 0.001) in the ALG20 and ALG40 groups compared to the CON and ALG60 ones (Table S3). Synergistetes were significantly increased (*p* = 0.012) in the microalgae-fed goats compared to the CON group (Table S3).

Figure 3A depicts the relative proportion of the sixteen most abundant (>0.1%) bacteria families within the goats’ rumen. Prevotellaceae, the most abundant family, tended to increase in the ALG40 group compared to the control (18.63 vs. 22.33%; *p* = 0.100) (Table S4). Succinivibrionaceae and Synergistaceae were increased significantly (*p* = 0.038 and *p* = 0.012, respectively) while Bacteroidaceae were decreased (*p* = 0.050) in the ALG20- and ALG40-fed goats compared to the CON (Table S4). Ruminococcaceae were significantly decreased (*p* = 0.011) in the microalgae-fed goats compared to the normally fed ones (Table S4). Eubacteriaceae, Clostridiales, Endomicrobiaceae, Erysipelotrichaceae, Atopobiaceae, and Eggerthellaceae were significantly decreased (*p* < 0.05) in the goats whose diets were supplemented with *Schizochytrium* spp., while Hungateiclostridiaceae tended to decrease (*p* = 0.089) in the ALG40 group compared to the CON group (Table S4). The methanogenic archaea family of Methanobacteriaceae tended to decrease (*p* = 0.077) in the ALG40 and ALG60 goats compared to the ALG20 group (Table S4).

At the genus level, a total of 578 genera was annotated. Among these genera, the 47 most abundant ones were statistically assessed (Table S5), and the first 20 (>0.3%) are presented as the relative proportion in Figure 3B. Following the trend of their family level, *Prevotella* species tended to increase in the ALG40-fed goats, while *Ruminococcus* were decreased significantly (*p* = 0.026) in the microalgae-fed goats compared to the CON goats (Table S5). *Fretibacterium* were increased (*p* = 0.012), while *Hungateiclostridium* were decreased (*p* = 0.018) in the ALG20 and ALG40 rumen liquid compared to the CON (Table S5). *Ruminobacter* species showed a significant increase (*p* = 0.049) in the ALG40 rumen fluid compared to the ALG60 and CON ones (Table S5). *Flintibacter*, *Intestinimonas*, *Eubacterium*, *Sporobacter*, *Neglecta*, *Blautia*, *Endomicrobium*, and *Stomatobaculum* were decreased significantly (*p* < 0.05) in the goats whose diets were supplemented with *Schizochytrium* spp. compared to the normally fed goats (Table S5). *Anaerovibrio* showed a significant increase (*p* = 0.049) in the ALG20 group compared to the CON and ALG60 groups (Table S5). A tendency for increase (*p* = 0.074) was observed in *Methanomicrobium* in the ALG60 group compared to the other dietary treatments including the CON (Table S5).

### 3.4. Relative Abundance of the Predominant Bacteria Species in the Goats’ Rumen

Figure 4A depicts the abundances of the goats’ rumen species featuring a pivotal role in protein, amino acid, and peptide degradation without ruling out their involvement in other functions, while Figure 4B describes those variables’ ability to correctly classify the dietary treatment. *Prevotella ruminicola* was found to be the dominant Bacteroidetes in the goats’ rumen. *Prevotella brevis* tended to increase (*p* = 0.097) in the ALG60 group compared to the CON (Table S6). Since no considerable fluctuations were observed at the species level, only a tendency for discrimination (*p* = 0.098; Wilks’s λ = 0.110) was found (Figure 4B). Specifically, the ALG20 group was located in the upper right corner of the plot away from the CON group (Function 1).

Figure 5A summarized the species with pronounced amylolytic activity. *Ruminobacter amylophilus*, prominent amylolytic bacteria within the rumen, were significantly increased in the ALG40-fed goats compared to the CON and ALG60 ones (Table S6). *Succinivibrio dextrinosolvens* also showed a significant (*p* = 0.044) enhancement in the ALG20 and ALG40 groups, while *Fretibacterium fastidiosum* was increased (*p* = 0.012) in the microalgae-fed goats compared to the CON group (Table S6). Due to the aforementioned alterations, the CON group was significantly different from the ALG20 and ALG40 groups in Function 1 (*p* = 0.012; Wilks’s λ = 0.327). However, a 60% correct classification was achieved.

Figure 6A summarized the overall reduction of cellulolytic species in the microalgae-fed rumen fluid. The dominant cellulose degradation species, *Ruminococcus flavefaciens*, was decreased (*p* = 0.016) significantly in the ALG40 group compared to the CON and ALG60 groups (Table S6). *Treponema bryantii* was considerably (*p* < 0.001) decreased in the *Schizochytrium* spp.-supplemented goats, while *Ruminococcus gauvreauii* and *Ruminococcus albus* tended to decrease (*p* = 0.070 and *p* = 0.099, respectively) in the ALG40 group compared to the CON (Table S6). These variations resulted in significant discrimination of the dietary treatments according to the most abundant cellulolytic bacteria (Figure 6B). Specifically, the CON group was mapped on the right-hand side of the plot, clearly distanced from the ALG20 and ALG40 groups for Function 1 (*p* < 0.001; Wilks’s λ = 0.115). Overall, 77.4% of the variables were correctly classified, while the CON variables achieved a higher classification score (90%).

Figure 7A presents a group of rumen bacteria with a key role in nutrients degradation and utilization. *Anaerovibrio lipolyticus* were increased significantly (*p* = 0.049) in the ALG20-fed goats, while *Intestinimonas butyriciproducens* and *Flintibacter butyricus* were decreased (*p* < 0.001) in the microalgae-fed goats compared to the CON group (Figure 7A; Table S6). Additionally, *Endomicrobium proavitum* were significantly increased in the rumen fluid of the goats fed with microalgae compared to the CON group (*p* = 0.009) (Table S6). *Succiniclasticum ruminis* and *Herbinix luporum* tended to increase in the ALG20 and ALG40 groups, respectively, compared to the CON group (*p* = 0.098; *p* = 0.068) (Table S6). Another important bacterium showing a significant reduction in the microalgae-fed goats was *Sporobacter termitidis* (*p* = 0.003) (Table S6). On the other hand, *Desulfovibrio desulfuricans* were significantly increased in the ALG20 and ALG40 groups compared to the CON group (*p* = 0.049) (Table S6). Figure 7B depicts the total abundance of Euryarchaeota species that were identified in the goats’ rumen. *Methanobrevibacter olleyae* were significantly decreased (*p* < 0.001) in the microalgae-fed goats compared to the CON group, while *Methanomicrobium mobile* tended to increase (*p* = 0.077) in the ALG60 group (Table S6). *Methanosphaera stadtmaniae*, *Methanobrevibacter millerae*, and *Methanobrevibacter thaueri* decreased only numerically (*p* > 0.10) in the ALG40-fed goats compared to the CON group (Table S6). Considering the sum of the *Methanobrevibacter olleyae*, *Methanosphaera stadtmaniae*, *Methanobrevibacter millerae*, and *Methanobrevibacter thaueri* abundances, a tendency for decrease (*p* = 0.095) was observed in the ALG40 goats.

### 3.5. Enzyme Activities, Ammonia Concentration, and Ruminal pH

**Figure 8:**
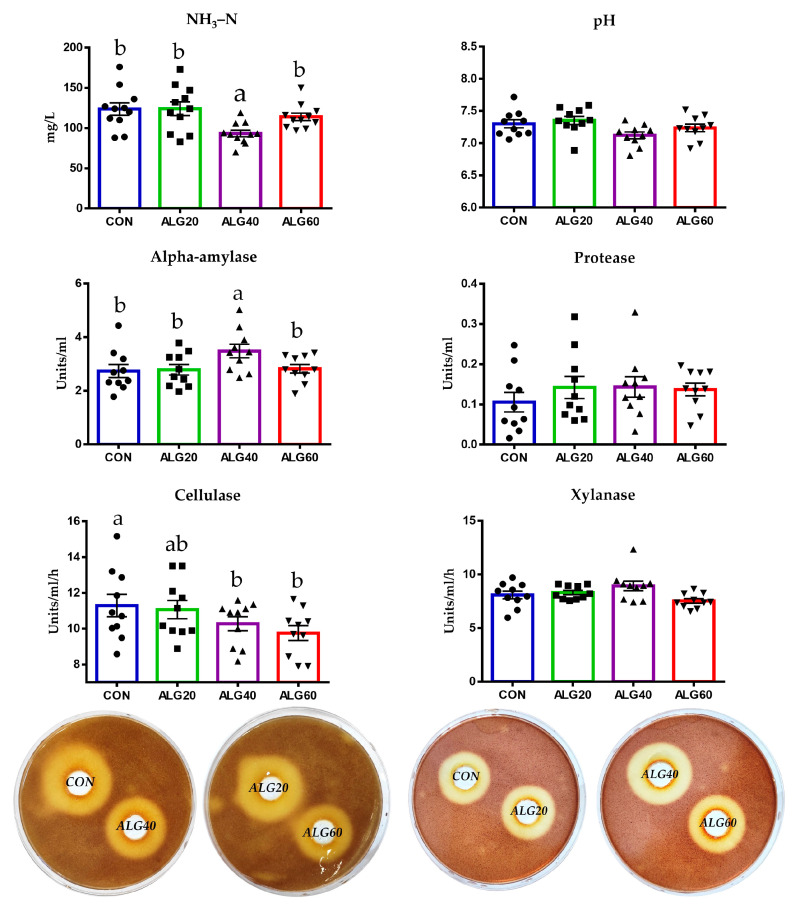
Effects of supplementing with microalgae *Schizochytrium* spp. on pH, ammonia concentration, alpha-amylase, protease, cellulase, and xylanase activity in the rumen fluid for the four dietary treatments (CON, ALG20, ALG40, and ALG60) within the two sampling times (20th and 40th days) illustrated in bar graphs ± SEM. Alpha-amylase and protease were determined spectrophotometrically, while cellulase and xylanase activities were assayed (individually) using Petri dishes. Petri dishes depict the area of the zone of clearing of the pooled rumen fluid of each dietary treatment which runs as a preliminary trial prior to the individual ones. Bars with different superscripts (a, b) between dietary treatments differ significantly (*p* ≤ 0.05) according to the analysis of variance (ANOVA) using a general linear model for repeated measures and post hoc analysis was performed when appropriate using the Tukey’s multiple range test. CON = control concentrate without microalgae (*Schizochytrium* spp.); ALG20 = CON with 20 g/Kg *Schizochytrium* spp.; ALG40 = CON with 40 g/Kg *Schizochytrium* spp.; ALG60 = CON with 60 g/Kg *Schizochytrium* spp.

Ammonia concentration was significantly decreased in the ALG40-fed goats compared to the other groups including the CON (Figure 8 and Table S8).

In detail, ammonia was determined to be 25% lower in the ALG40 group compared to the CON group (*p* = 0.008) (Table S8). Ruminal pH did not differ significantly (*p* > 0.05) between the dietary treatments (Table S8). Alpha-amylase activity was increased in the ALG40 group compared to the other dietary treatments (Figure 8; Table S8). More specifically, an increase of 27% was found in the ALG40 group compared to the CON group (*p* = 0.048) (Table S8). Protease activity portrayed an upward trend in the microalgae-fed groups; however, the enhancement was not significantly considerable (*p* = 0.456; Figure 8; Table S8). Cellulase and xylanase activities were determined by measuring the zones of clearance of the rumen inoculum in the cellulose and xylan agar Petri dishes, respectively. Cellulase activity was decreased (*p* = 0.050) in the ALG40 and ALG60 groups compared to the CON group, while the ALG20-fed goats were not affected significantly. Xylanase activity was not considerably different (*p* = 0.356) (Table S8).

### 3.6. The Correlation between Ruminal Bacteria and Milk Composition

**Figure 9:**
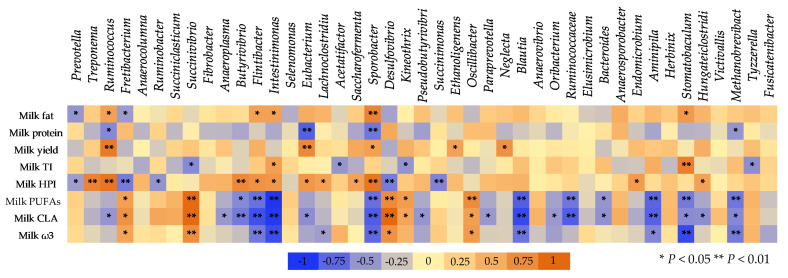
Spearman’s correlation between the most abundant genera within the goats’ rumen and milk performance and its important constituents. Milk fat expressed as percentage, milk protein expressed as percentage, milk yield expressed as ml/day; milk TI (thrombogenic index) = (C14:0 + C16:0 + C18:0)/(0.5 × MUFA) + (0.5 × ω6 PUFA) + (3 × ω3 PUFA) + (ω3 PUFA/ω6 PUFA); milk HPI (health-promoting index) = (ω6 PUFA + ω3 PUFA + MUFA)/(C12:0 + 4 × C14:0 + C16:0); Milk PUFAs (polyunsaturated fatty acids) = cis-9, trans-11 C18:2 + C18:2n−6c + C18:2n−6t + C18:3n−3 + C18:3n−6 + C20:3n−3; milk CLA (cis-9, trans-11 C18:2); and milk ω3—the sum of C18:3n−3, C20:3n−3, and C22:6n−3.

Figure 9 presents the Spearman’s correlation between the dominant microbial genera in the goats’ rumen with the goats’ milk performance and its pivotal constituents. Milk fat was negatively correlated (*p* < 0.05) with the abundance of *Prevotella* and *Fretibacterium* species, while a positive correlation was found with *Ruminococcus*, *Flintibacter*, *Intestinimonas*, and *Sporobacter*. Milk protein was negatively correlated with *Ruminococcus* (*p* < 0.05), *Eubacterium* (*p* < 0.01), *Sporobacter* (*p* < 0.01), and *Methanobrevibacter* (*p* < 0.05) abundances. Positive correlations were observed between milk yield and *Ruminococcus* (*p* < 0.01), *Eubacterium* (*p* < 0.01), *Sporobacter* (*p* < 0.05), *Ethanoligenens* (*p* < 0.05), and *Neglecta* (*p* < 0.05) abundances. The health-promoting index (HPI) which used to evaluate the potential nutritional value of milk was negatively correlated with *Prevotella* (*p* < 0.05), *Fretibacterium* (*p* < 0.01), *Succinimonas* (*p* < 0.01), and *Ruminobacter* species (*p* < 0.05), while positive correlation was observed with *Treponema* (*p* < 0.01), *Ruminococcus* (*p* < 0.01), *Butyrivibrio* (*p* < 0.01), *Flintibacter* (*p* < 0.05), *Intestinimonas* (*p* < 0.05), *Eubacterium* (*p* < 0.05), *Lachnoclostridium* (*p* < 0.05), *Saccharofermenta* (*p* < 0.05), *Endomicrobium* (*p* < 0.05), *Hungateiclostridium* (*p* < 0.05)**, and *Sporobacter* (*p* < 0.01).

Milk polyunsaturated fatty acids (PUFA) were negatively correlated with *Butyrivibrio* (*p* < 0.05), *Flintibacter* (*p* < 0.01), *Intestinimonas* (*p* < 0.01), *Sporobacter* (*p* < 0.01), *Blautia* (*p* < 0.01), Ruminococcaceae (*p* < 0.01), *Bacteroides* (*p* < 0.05), *Aminipila* (*p* < 0.01), *Stomatobaculum* (*p* < 0.01), and *Methanobrevibacter* (*p* < 0.01). On the contrary, positive correlation was found with *Fretibacterium, Succinivibrio, Desulfovibrio, Kineothrix*, and *Oscillibacter*. Milk conjugated linoleic acid showed negative correlation with *Ruminococcus* (*p* < 0.05), *Anaeroplasma* (*p* < 0.05), *Butyrivibrio* (*p* < 0.01), *Flintibacter* (*p* < 0.01), *Intestinimonas* (*p* < 0.01), *Eubacterium* (*p* < 0.05), *Sporobacter* (*p* < 0.01), *Pseudobutyrivibrio* (*p* < 0.05), *Paraprevotella* (*p* < 0.05), *Blautia* (*p* < 0.01), *Oribacterium* (*p* < 0.05), Ruminococcaceae (*p* < 0.01), *Bacteroides* (*p* < 0.05), *Aminipila* (*p* < 0.01), *Hungateiclostridium* (*p* < 0.05), and *Methanobrevibacter* (*p* < 0.05). In contrast, CLA and total milk ω3 fatty acids were positively correlated with *Fretibacterium* (*p* < 0.05), *Succinivibrio* (*p* < 0.01), *Desulfovibrio* (*p* < 0.05), and *Oscillibacter* (*p* < 0.05).

## 4. Discussion

Rumen habitat is governed by complex metabolic interactions between microbes and subsequently also with their animal host in order to facilitate and optimize the utilization of the fed materials into high-value nutrients. Metagenomics has been proven to be a valuable tool for predicting and orchestrating nutrient utilization achieved through novel biotechnological dietary interventions.

This work aimed to contribute to a collective effort of understanding rumen microbiome modulations under the impact of supplementing PUFA-rich marine microbe (*Schizochytrium* spp.) in a goats’ diet by bridging the current microbiome screening techniques and credible biochemical assays. Our preliminary observations under the same experimental conditions in a narrow rumen microbiome spectrum are now greatly validated in the realms of the present study indicating a strong impact of *Schizochytrium* spp. in the goats’ rumen habitat [9,10].

### 4.1. Tight Linkage between the Rumen Microbiome and the Goats’ Performance

The richness and diversity of the ruminal bacterial microbiota are important indicators of normal rumen biochemical processes. In our study, the lower Shannon index at the species level in the ALG40 group suggested that the bacteriome composition was modulated and tended to be less diverse in the ALG40-fed goats. Notably, Zeng et al. [34] reported a less diverse rumen bacteria community in milk fat-depressed (MFD) cows due to an experimental diet with high starch content. Thus, it is plausible to assume that changes in key microbes and fermentation pathways within the rumen could induce a cascade of holistic modifications at the animal performance level.

Moreover, the reduction of concentrate intake in the ALG60 group resulted in an altered forage-to-concentrate ratio (60:40 instead of 50:50), which may further govern bacteria populations within the goats’ rumen. Despite the ALG60 goats’ lower feed intake, their milk yield and body weight were not affected significantly, suggesting a more efficient feed utilization [7]. In the present study, Firmicutes suppression was accompanied by a tendency for Bacteroidetes enhancement, while at the same time, MFD occurred in the ALG40 and ALG60 groups [7]. Interestingly, Delgado et al. [35] reported that the most efficient cows had larger relative abundances of Bacteroidetes and *Prevotella* and a lower relative abundance of Firmicutes. In context, Jami et al. [36] observed a positive correlation between the rumen Firmicutes-to-Bacteroidetes ratio and milk fat content. A similar trend was further observed on the relative abundance of Bacteroidetes that remained unaffected, while Firmicutes showed a tendency to decrease with the dietary supplementation with 20.8 g *Schizochytrium* spp./day in MFD ewes [6,37]. In our work, *Prevotella*, the dominant genus of Bacteroidetes, was found to be negatively correlated with milk fat content, further supporting the link between rumen microbiome composition and productive characteristics of goats.

### 4.2. The Biohydrogenation Process and the Bacteria Species Involved Remain Unbridgeable

Biohydrogenation appears to be the central rumen function for strategies aiming to enrich ruminants’ products with PUFAs. *Butyrivibrio* species are considered to be the main contributors to biohydrogenation and thus have been extensively studied [38,39]. However, the paradox of many relative studies is finding the extent of accumulated biohydrogenation intermediates accompanied by inhibition of C18:0 formation without major alterations of *Butyrivibrio* abundances [16], forcing researchers to correlate other genera with the rumen biohydrogenation process. Our results are in agreement with several previous studies in which biohydrogenation occurred while *Butyrivibrio* remained unaffected in response to DHA supplementation [16,40,41,42] (Table S9). Thus, it could be hypothesized that other strains might play a role in the final biohydrogenation step. Such strains may belong to the Lachnospiraceae family and, more specifically, to the *Blautia* genus, as previously shown [16]. Indeed, in our study, *Flintibacter*, *Intestinimonas*, *Sporobacter*, and *Blautia* species were more tightly correlated with milk ω3, PUFA, and CLA content than *Butyrivibrio* species, pointing to direct or indirect involvement. However, DNA sequencing is unable to provide a reliable footprint from metabolically active bacteria. In our and other previous studies, the correlation of the rumen-accumulated intermediates with the DNA footprint instead of transcript profiling may be the substantial factor we struggle to reliably bridge in our assumptions [43].

Above and beyond, *Fretibacterium* has also been reported to be involved in biohydrogenation of C18:2n6c [44]. Our results are partially in accordance with the previous observations regarding *Fretibacterium* biohydrogenation properties since C18:2n6c was decreased in the rumen fluid of microalgae-treated goats as well [10] (Table S9). However, the final product of C18:2n6c biohydrogenation (C18:0) was significantly lower, while biohydrogenation intermediates (trans-11 C18:1) were accumulated in the rumen of microalgae-fed goats [10] (Table S9).

The ratio of DHA to DPA in *Schizochytrium* spp. was 3.2 (21.8/6.8; Table 1), while in the rumen fluid, it ranged between 1.3 to 1.8 (Table S9), indicating that DHA was biohydrogenated to a larger extent compared with DPA. In accordance with our findings, Toral et al. [45] observed a several-fold higher transfer efficiency of DPA than that of DHA in algae-fed ewes. Unfortunately, the literature is lacking for enzymes that may regulate the DHA hydrogenation pathway in the rumen [46], while the degradation of DHA and DPA to other isomers is also hard to study in in vivo trials due to their short lifetime [47].

### 4.3. Microbiome Shifting Reflects Alterations in Rumen Biochemistry

Proteobacteria enhancement in the rumen has been characterized as an unfavorable condition (also known as “dysbiosis”) which has been associated with lower ruminal pH [48]. Interestingly, in our work, ruminal pH was not affected by microalgae supplementation in goats’ diets, assuming that the rumen habitat was not a substantial factor for Proteobacteria increase. It could be hypothesized that the mechanism underlying Proteobacteria escalation may be attributed to their opportunistic properties [49]. Approximately 85% of Proteobacteria constitute the Succinivibrionaceae family which includes several amylolytic and saccharolytic species [50]. More specifically, Succinivibrionaceae members commonly produce succinate, the precursor of propionate, a pivotal energy supplier for the host [50]. Intriguingly, bacterial groups associated with the production of propionate precursors such as the Succinivibrionaceae family depict a negative correlation with CH_4_-emitting individuals [18]. As such, it is hypothesized that the upsurge of Succinivibrionaceae may partially inhibit methane formation through H_2_ competition since methyl-coenzyme M reductase (MCR) expression is regulated by H_2_ availability in the rumen [18]. *Succinivibrio dextrinosolvens*, Gram-negative, curved, rod-shaped bacteria that degrade starch and produce succinate, acetate, and formate, have been associated with high methane-producing individuals [51]. More specifically, it has been reported that *S. dextrinosolvens* stimulate methane formation through Methanomassiliicoccales in in vitro cocultures [51]. In our previous studies, the Methanomassiliicoccales relative abundance was decreased in the rumen solid fraction of microalgae-fed goats [9] while tending to decrease with dietary supplementation with 20 and 40 g *Schizochytrium* spp. in the rumen liquid [10]. This set of evidence indicates that the inclusion of *Schizochytrium* spp. Either disrupts the aforementioned bacteria dependence or signifies the discrepancies between in vitro and in vivo trials. However, the assessment of methanogens per se was out of the scope of the present study as their appropriate hypervariable region for amplification is considered to be the V6–V8 region of 16S rRNA [48,52]. Even though our platform (V3–V4) provided data concerning species that are ordered into the Euryarchaeota phylum, in a few cases, methanogen abundances were found to be below the detection level, making it hard to generate dependable assumptions regarding their response to dietary supplementation. Nevertheless, the abundance of selected methanogenic microbes has been previously reported in the particle-associated microbiota [9] and floated in the rumen liquid [10] of the goats that were supplemented with *Schizochytrium* spp. under the same experimental conditions.

In our results, total cellulolytic activity was found to be significantly lower in the ALG40 and ALG60 goats, correlating well with the observed reduction of fibrolytic bacteria. In vitro studies have reported the inhibitory effect of marine origin PUFAs on cellulolytic bacteria [12,15]. However, there is a serious gap between the turning-point supplementation level and its impact on animal performance. Our results suggested that supplementing 20 g of microalgae *Schizochytrium* spp. did not compromise the fibrolytic potential within the goats’ rumen despite the overall trend that was observed. The animals’ performance remained unaffected, whilst milk was significantly enriched with ω3 fatty acids in response to 20 g *Schizochytrium* spp. [7]. Alpha-amylase activity was increased in the ALG40 goats, while protease activity was increased only numerically in the microalgae treatments despite the enhancement of some *Prevotella* species in the ALG40 group. Overlooking the apparent proteolytic tendency, NH_3_–N concentration in the ALG40 group was found to be significantly lower. In summary, an ammonia proportion may be utilized by rumen microorganisms for cell growth in the presence of available energy in the form of sugars due to a higher amylase activity [53].

Recently studied bacteria *Endomicrobium proavitum* are reported to contain an unusual nitrogenase (belonging to group IV) capable of reducing feed nitrogen to ammonia [54]. In our study, the reduction of the *E. proavitum* population in the microalgae-fed goats may conceal an improved N efficiency. It is worth mentioning at this point that another substantial factor that may be responsible for ammonia suppression in the ALG40 group could be attributed to rumen protozoa. More specifically, rumen ammonia concentration has been found to be considerably decreased in defaunated (removal of protozoa) ruminants due to the exclusion of protozoal proteolytic and deaminative enzymes [55]. However, protozoa and especially *Entodinium* spp. relative abundance in the rumen of microalgae-fed goats was not altered based on our previous results [9,10], ruling out the effect of protozoa on the altered ammonia concentration. Thus, considering the aforementioned, we could speculate that any improvement in nitrogen utilization in the event of *Schizochytrium* spp. inclusion was not caused at the expense of protozoal fibrolytic activity.

Bacteriome requires a vast amount of energy which usually equates to the maintenance of its basic functions [56]. Thus, energy in the form of high-fermentable sugars and short-chain fatty acids is the restrictive factor for optimal microbial growth [56]. Taking into account that microbial proteins supply 60–85% of the amino acids that reach ruminants’ intestines, a potential enhancement of microbial proteins in the ALG40 group appears to portray a crucial response of the rumen ecosystem towards microbiome disturbance in order to preserve animal performance. Nevertheless, it remains an open question whether the positive alliance between amylolytic and proteolytic potential is a sufficient and sustainable approach to substitute for compromised fibrolytic activity.

Another metabolic function that could have contributed to maintaining the animals’ performance despite cellulolysis suppression may relate to a cross-feeding interaction between bacteria and the host animal. More specifically, due to the high metabolic fermentation of starch in the ALG40 group, the available energy in bacteria may be utilized to form glycogen and other carbohydrate compounds in a process named “reserve carbohydrate synthesis” which is activated in the event of energy excess within the rumen [56]. In summary, medium and high levels of *Schizochytrium* spp. suppressed the overall cellulolytic activity through the fibrolytic bacteria toxicity, whilst amylolytic and partially the proteolytic bacteria grasped the opportunity to expand their niche as a result of the general reduction in the numbers of fibrolytic bacteria.

*Eubacterium coprostanoligenes* ferment arabinose, cellobiose, fructose, glucose, mannose, and melibiose into acetic, formic, and succinic acids and hydrogen. In agreement with our findings, Popova et al. [57] found a lower abundance of *E. coprostanoligenes* in the rumen of the cattle that were fed a diet with a high lipid content. *Sporobacter termitidis*, an isolated species from the intestines of wood-feeding termites, have been reported to be involved in fiber degradation within the rumen [58]. Furthermore, recent studies signify the potential role of *S. termitidis* in producing large amounts of CH_4_ [58].

Another important amylolytic bacteria, *Fretibacterium fastidiosum*, may further contribute to the increased amylase activity [59]. Unlike other amylolytic bacteria, *Ruminococcus bromii* populations were negatively affected by *Schizochytrium* spp. inclusion. Taking into consideration that *Ruminococcus bromii* is a Gram-positive species [60] whilst the majority of the rest of the amylolytic bacteria within the rumen consist of Gram-negative strains, these fluctuations may be attributed to LCPUFAs’ toxic effect on the membranes of Gram-positive bacteria [11]. More specifically, PUFAs’ double bonds alter the shape of the molecule [61]; the kinked unsaturated fatty acids disrupt the lipid bilayer structure inducing ion leakage and/or decoupling intramembrane pathways resulting in chemiosmotic difficulties [62]. Another explanation of PUFA toxicity may be related to metabolic imbalances in acyl CoA metabolism [62]. Remarkably, recent evidence subverts the aforementioned assumptions, indicating that unsaturated fatty acids did not considerably affect bacterial growth of both Gram-negative and -positive strains [63], whilst unsaturated fatty acids are involved in the prevention of biofilm formation in Gram-positive bacteria, even at the lowest treated level [63]. Hence, rumen bacteria populations could return to a planktonic lifestyle if the biofilm is dispersed, making them prone to abiotic factors.

*Herbinix luporum* constitutes an exception based on our observations, showing an upward tendency in the ALG40 group despite its classification as a Gram-positive strain. However, in terms of the physiological perspective, *H. luporum* may be responsible for xylanase escalatory tendency in the ALG40 rumen fluid since it is able to digest cellulosic and hemicellulosic substrates, with the highest activity found to occur on xylan [64]. A wide range of xylanases is also produced by the dominant rumen genus, *Prevotella*, the relative abundance whereof tended to increase in the ALG40-fed goats [65]. Thus, despite the suppression of cellulolytic bacteria which also exerts hemicellulolytic properties, xylanase activity was not suppressed in the microalgae-fed goats, probably due to the hemicellulolytic potential of *Prevotella*. This equilibration between cellulase and xylanase activities may play a pivotal role in the overall rumen fibrolysis.

### 4.4. Difficulties of Farm-Scale Implementation and Future Perspectives

Passing over the sustainability issues that could be unveiled through a compromised cellulolytic ability, our study reported an important warning flag. *Desulfovibrio desulfuricans*, sulfate-reducing rumen bacteria, were found in higher abundances in the ALG20 and ALG40 groups. The formed hydrogen sulfite is released into the atmosphere through eructation; however, H_2_S is quickly absorbed through the intestinal wall making animals sensitive to its toxicity. *D. desulfuricans* enhancement may be attributed to the decline of an interactive and competitive relationship between methanogens and sulfate-reducing bacteria (SRB) for H_2_. More specifically, both SRB and methanogens compete with each other for the requirement of H_2_ to reduce sulfate to sulfide and of CO_2_ to CH_4_, respectively [66]. Corroborating this conclusion, in our previous work [9], methanogenic archaea adhering to feed particles were considerably suppressed in goats that were fed with *Schizochytrium* spp. under the same experimental conditions. Thus, the accumulated H_2_ may be utilized as a substrate for hydrogen sulfide reduction. *D. desulfuricans* were not significantly affected in the ALG60 group, probably due to the modified concentrate-to-forage ratio. Particularly, Morine et al. [67] reported that the level of H_2_S in the rumen decreased linearly with the upsurge in the dietary roughage neutral detergent fiber (rNDF) content. Overall, the increase of *D. desulfuricans* may result in an accumulation of hydrogen sulfite in the goats’ rumen, potentially concealing dangerous consequences for animals’ health and performance which may be eliminated by altering the forage-to-concentrate ratio in *Schizochytrium* spp.-fed goats [68]. In this context, *Schizochytrium* spp. inclusion in goats’ diets may be accompanied by reductive acetogenic bacteria in order to investigate their ability to counteract the competition for H_2_ utilization.

Marine origin PUFAs are found to exert toxic effects on the Prevotellaceae family and on the *Ruminococcus* and *Succinivibrio* species as well in cows and sheep [15]. However, in vitro trials using pure cultures have indicated that DPA alone does not considerably affect the aforementioned taxa compared to DHA inclusion [15]. It should also be noted that DPA shows a higher apparent transfer efficiency from feed to milk than DHA (13% vs. 9%) [7], while Toral et al. [45] reported an even higher proportion. Therefore, DPA constitutes the most efficient biomolecule amongst those of marine origin to enrich dairy products with bioactive compounds without inducing severe unfavorable alterations in key bacteria communities within the rumen. Microalgae appear to be a customized source of PUFAs (targeting the increase of DPA) either by regulating gene expressions [69] or through cultivating manipulations [70]. Taking the above into consideration, targeted rumen microbiome engineering could be achieved after understanding the rumen microbiome and its complex biochemical procedures, provided that biotechnological bioactive compounds such as tailor-made microalgae could be produced.

Till recently, it has been vastly acknowledged that PUFA-enriched diets induce several changes within the rumen microbiome, the primary one being cellulolysis suppression. Previously published data correlated with compromised animal performance and therefore seemed discouraging for further investigation. In our study, bridging the novel molecular techniques (NGS) with such a set of targeted biochemical assays and involving the simultaneous investigation of different (optimal) dietary supplementation levels, it was indicated that both the rumen ecology and the host are characterized by outstanding plasticity with the ultimate goal of preserving animals’ performance. Apparently, it is unsustainable to exploit this plasticity for a long time under farm-scale conditions; however, further studies are required to clarify if we could harness microbiome plasticity for short-term treatments, focusing on functional dairy products and methane mitigation strategies without affecting the overall farm sustainability.

## 5. Conclusions

Although the cellulolytic potential was substantially suppressed, the proteolytic and amylolytic functions tended to be enhanced, indicating an adaptation mechanism in order to preserve lactation in the medium inclusion level (40 g *Schizochytrium* spp.). Generally, alternate beneficial modulations were observed related to functional end products and environmental impact; at the same time, warnings flags arose regarding the animal performance and balanced homeostasis. In this context, future rumen microbiome engineering should not only confirm the aforementioned observation in the metabolic spectrum (transcriptomic and proteomic approaches), but also evaluate the synergistic potential of several feed additives to counteract the arising challenges.

## Figures and Tables

**Table 1:** Alfalfa hay, DHAgold™, and concentrate mix: chemical composition (g/kg) and fatty acids profile (g/100 g total fatty acids).

**Chemical composition of feeds (g/kg)**						
	Alfalfa hay	DHAgold™	CON	ALG20	ALG40	ALG60
Dry matter	927	980	922	927	918	926
Crude protein	134	167	126	127	127	127
Crude fat	13	556	43	57	63	79
Crude fiber	319	45	50	52	52	56
NDF	503	-	157	152	149	169
ADF	381	-	61	56	57	64
Ash	72	88	92	95	93	89
**Fatty acid composition (g/100g total FA)**						
C_14:0_	2.3	5.9	0.1	1.4	2.8	3.7
C_16:0_	31.6	13.1	14.3	15.2	17.1	18.7
C_18:0_	5.8	0.3	3.9	3.2	2.7	2.5
C_18:1 cis−9_	11.6	n.d.	36.1	29.8	24.2	21.8
C_18:2 n−6_	26.4	n.d.	42.2	36.5	31.1	28.6
C_18:3 n−3_	19.7	0.1	1.7	1.6	1.3	1.2
C_22:5 n−6_	n.d.	6.8	n.d.	2.9	5.3	6.2
C_22:6 n−3_	n.d.	21.8	0.1	7.3	13.4	15.5

NDF = neutral detergent fiber; ADF = acid detergent fiber.

## Data Availability

The data are contained within the article and the Supplementary Materials.

## Supplementary Materials

The following are available online at https://www.mdpi.com/article/10.3390/microorganisms9071528/s1, Table S1: Libraries concentrations measured with Picogreen; Table S2: Number of sequences, average length, total Mb and average quality after trimming; Table S3: Relative abundances of the identified phyla, unmapped bacteria, and non-clustered sequences (no-hits) in the liquid of the rumen for the four dietary treatments (CON, ALG20, ALG40, and ALG60) on the two sampling points (20th and 40th experimental days); Table S4: Relative abundances of the identified families in the liquid of the rumen for the four dietary treatments (CON, ALG20, ALG40, and ALG60) on the two sampling points (20th and 40th experimental days); Table S5: Relative abundances of the most abundant bacteria genera (>0.06%) and methanogenic archaea in the liquid of the rumen for the four dietary treatments (CON, ALG20, ALG40, and ALG60) on the two sampling points (20th and 40th experimental days); Table S6: Relative abundances of the most abundant bacteria species and methanogenic archaea in the liquid of the rumen for the four dietary treatments (CON, ALG20, ALG40, and ALG60) on the two sampling points (20th and 40th experimental days); Table S7: Ingredients of the concentrate (g/kg), average feed consumption (g/goat/day), intake of nutrients and fatty acids (g/goat) of the four diets; Table S8: Effects of supplementing with microalgae *Schizochytrium* spp. on pH, ammonia concentration, alpha-amylase, protease, cellulase, and xylanase activity in the liquid of the rumen for the four dietary treatments (CON, ALG20, ALG40, and ALG60) on the two sampling points (20th and 40th experimental days); Table S9: The mean individual fatty acids (FA) (% of the total FAs) of the goats’ rumen fluid for the four dietary treatments (CON, ALG20, ALG40, and ALG60) on the two sampling points (20th and 40th experimental days); Table S10: The mean individual fatty acids (FA) (% of the total FAs), FA groups, SFA/UFA and ω6/ω3 ratios, and Δ−9 desaturate indexes of milk from the goats fed diets with different levels (g/kg concentrate) of microalgae *Schizochytrium* spp. (CON, ALG20, ALG40 and ALG60) on the two sampling points (20th and 40th experimental days); Figure S1: Rarefaction curves of the bacterial population at the genus taxonomic level for 16S rRNA sequencing; Figure S2: Validation of the 16S rRNA sequencing (IL) by qPCR (PCR) in terms of the relative abundances of *Prevotella ruminicola*, *Ruminobacter amylophilus*, *Fibrobacter succinogenes*, and *Butyrivibrio fibrisolvens*. The microalgae-treated groups (ALG20, ALG40, and ALG60) of each method (IL vs. PCR) were expressed relatively as fold changes towards the control (CON) group in order to be similarly visualized. The data are depicted as trends and no statistic was applied; Figure S3: Relative abundances of the identified phyla, unidentified clusters, and no-hit sequences for the four dietary treatments (CON, ALG20, ALG40, and ALG60) within the two sampling times (20th and 40th days) illustrated in the cumulative bar graph; Figure S4: Discriminant plots separating (A) the four dietary treatments (CON, blue ○; ALG20, green □; ALG40, purple △; and ALG60, red ▽) according to the pooled data of the two sampling times (20th and 40th experimental days) entered together as independents on the explained abundances of A) the fourteen identified phyla and B) the thirty-four identified families in the goats’ rumen. In the (A) plot, the proportion of the samples that were correctly classified was 80%, while the Wilks’s λ was observed at 0.067 for Function 1 (*p* < 0.001) and 0.291 for Function 2 (*p* = 0.075). In the (B) plot, the proportion of the samples that were correctly classified was 82.5%, while the Wilks’s λ was observed at 0.115 for Function 1 (*p* = 0.013) and 0.429 for Function 2 (*p* = 0.496). CON = control concentrate without microalgae (*Schizochytrium* spp.); ALG20 = CON with 20 g/kg *Schizochytrium* spp.; ALG40 = CON with 40 g/kg *Schizochytrium* spp.; ALG60 = CON with 60 g/kg *Schizochytrium* spp.
