# Supplementary material for: Changes in the Rumen Bacteriome Structure and Enzymatic Activities of Goats in Response to Dietary Supplementation with Schizochytrium spp
Source: Microorganisms. 2021 Jul 17;9(7):1528. doi: 10.3390/microorganisms9071528 (PMC8303384; doi:10.3390/microorganisms9071528)
Supplement: Supplementary file 1 [file microorganisms-09-01528-s001.zip › microorganisms-1286240-supplementary.pdf]

microorganisms

MDPI

Table S1. Libraries concentrations measured with Picogreen.

Sample barcode	Animal	ng/uL
193281	199A	80.24
193282	1340A	65.00
193283	1844A	86.09
193284	1717A	38.52
193285	1719A	77.64
193286	1711A	69.72
193287	1718A	49.84
193288	66A	39.54
193289	63A	44.11
193290	68A	45.64
193291	1259A	49.77
193292	965A	70.99
193293	100A	93.02
193294	1842A	84.88
193295	1846A	87.97
193296	1841A	40.65
193297	1713A	82.38
193298	1362A	18.40
193299	1257A	98.72
193300	1258A	47.41
193301	199B	64.32
193302	1340B	62.17
193303	1844B	98.65
193304	1717B	79.46
193305	1719B	93.65
193306	1711B	73.33
193307	1718B	107.97
193308	66B	34.87
193309	63B	63.05
193310	68B	64.08
193311	1259B	80.31
193312	965B	81.75
193313	100B	75.45
193314	1842B	44.98
193315	1846B	92.81
193316	1841B	89.55
193317	1713B	95.59
193318	1362B	87.76
193319	1257B	83.92
193320	1258B	75.71

Table S2. Number of sequences, average length, total Mb, and average quality after trimming.

Group	Animal Num.	Num. Seq	Av. Length	Total Mb	Av. Qual
CON	199	52868	403.52	21.33	37.51
	1340	48964	403.09	19.74	37.38
	1844	44267	405.16	17.94	37.44
	1717	51646	404.58	20.89	37.48
	1719	53744	400.61	21.53	37.37
ALG20	1711	57193	404.6	23.14	37.5
	1718	61984	403.99	25.04	37.46
	66	54085	405.63	21.94	37.45
	63	50066	407.55	20.4	37.46
	68	59904	403.54	24.17	37.5
ALG40	1259	52494	405.53	21.29	37.39
	965	53123	407.55	21.65	37.58
	100	47204	403.61	19.05	37.37
	1842	47633	403.97	19.24	37.3
	1846	43895	395.29	17.35	37.19
ALG60	1841	49339	405.16	19.99	37.36
	1713	47898	399	19.11	37.19
	1362	47504	403.64	19.17	37.47
	1257	91883	399.51	36.71	37.26
	1258	48478	402.26	19.5	37.36
CON	199	51667	402.48	20.79	37.22
	1340	51613	402.49	20.77	37.28
	1844	49443	402.54	19.9	37.32
	1717	54387	403.88	21.97	37.46
	1719	51376	402.72	20.69	37.44
ALG20	1711	52586	403.93	21.24	37.37
	1718	117337	400.75	47.02	37.25
	66	50358	405.21	20.41	37.5
	63	49665	404.43	20.09	37.46
	68	57118	405.53	23.16	37.4
ALG40	1259	50855	404.44	20.57	37.27
	965	56080	401.83	22.53	37.36
	100	51283	404.67	20.75	37.28
	1842	60596	406.08	24.61	37.47
	1846	51023	404.15	20.62	37.42
ALG60	1841	50358	407.2	20.51	37.61
	1713	47381	402.35	19.06	37.38
	1362	47890	402.67	19.28	37.35
	1257	49454	402.37	19.9	37.26
	1258	46574	402.42	18.74	37.39

Table S3. Relative abundance of identified phyla, unmapped bacteria, and no-clustered sequences (no-hit) in the liquid of rumen of the four dietary treatments (CON, ALG20, ALG40, and ALG60) on two sampling points (20th and 40th experimental day).

**Phylum**	**Treatment (T)**					**Sampling time (S)**			**Effect ‡**		
	CON	ALG20	ALG40	ALG60	SEM†	20	40	SEM†	T	S	T x S
Actinobacteria	0.125[a]	0.083[ab]	0.076[b]	0.055[b]	0.014	0.072[a]	0.096[b]	0.008	0.021	0.011	0.324
Bacteroidetes	18.86[t]	21.91	22.50[t]	18.92	1.360	19.89	21.19	0.875	0.099	0.272	0.046
Firmicutes	15.66[a]	12.35[b]	10.07[b]	11.19[b]	0.933	12.82	11.81	0.550	0.004	0.126	0.058
Chloroflexi	0.063[a]	0.179[b]	0.141[b]	0.222[b]	0.028	0.168	0.134	0.020	0.007	0.297	0.025
Elusimicrobia	0.387	0.147	0.271	0.327	0.058	0.197[a]	0.368[b]	0.035	0.056	0.001	0.079
Euryarchaeota	0.106	0.131	0.053	0.078	0.024	0.087	0.097	0.017	0.163	0.662	0.007
Fibrobacteres	1.642	1.792	1.441	1.186	0.269	2.151	0.879	0.151	0.439	<0.001	0.361
Fusobacteria	0.029	0.022	0.015	0.008	0.006	0.021	0.016	0.004	0.116	0.418	0.033
Lentisphaerae	0.162	0.179	0.121	0.174	0.051	0.164	0.154	0.035	0.850	0.788	0.123
Proteobacteria	2.437[a]	4.762[b]	5.576[b]	3.539[ab]	0.654	4.670	3.487	0.423	0.019	0.075	0.025
Spirochaetes	1.291	0.777	0.770	0.860	0.184	1.112[a]	0.736[b]	0.120	0.185	0.030	0.234
Synergistetes	1.133[a]	3.287[b]	3.288[b]	2.397[b]	0.454	2.280	2.772	0.277	0.012	0.116	0.461
Tenericutes	0.508	0.528	0.246	0.482	0.145	0.485	0.398	0.078	0.503	0.324	0.219
Verrucomicrobia	0.003	0.003	0.004	0.002	0.001	0.004	0.002	0.001	0.597	0.057	0.205
Unmapped	43.79	43.44	44.60	47.57	4.895	44.23	45.51	4.32	0.141	0.801	0.454
No-hits	13.82[a]	10.40[b]	10.83[b]	12.95[a]	0.368	11.65	12.35	0.265	<0.001	0.075	0.451

Means with different superscript (a, b, c, d) between dietary treatments and (a, b) between sampling time differ significantly ($p \leq 0.05$) while, t; trend refers to $p < 0.10$. CON = control concentrate without microalgae; ALG20 = control concentrate with 20 g/Kg *Schizochytrium* sp.; ALG40 = control concentrate with 40 g/ Kg *Schizochytrium* sp.; ALG60 = control concentrate with 60 g/ Kg *Schizochytrium* sp. ‡ Effect: The dietary treatment (T), time (S), and the interaction between dietary treatment x time (TxS) effects were analyzed by ANOVA using a general linear model (GLM) for repeated measures and Post hoc analysis was performed when appropriate using Tukey multiple range test. †SEM: Standard error of the mean.

Table S4. Relative abundance of identified families in the liquid of rumen of the four dietary treatments (CON, ALG20, ALG40, and ALG60) on two sampling points (20th and 40th experimental day).

**Family**	**Treatment (T)**					**Sampling time (S)**			**Effect ‡**		
	CON	ALG20	ALG40	ALG60	SEM†	20	40	SEM†	T	S	T x S
Prevotellaceae	18.629^{t}	21.737	22.332^{t}	18.756	1.357	19.710	21.017	0.878	0.100	0.270	0.046
Lachnospiraceae	4.157	3.896	3.111	3.386	0.357	3.610	3.665	0.217	0.194	0.847	0.101
Succinivibrionaceae	1.927^{a}	3.704^{bc}	4.769^{c}	2.750^{ab}	0.648	3.885	2.691	0.423	0.038	0.080	0.029
Ruminococcaceae	3.838^{a}	1.910^{b}	1.513^{b}	2.212^{b}	0.449	2.681^{a}	2.055^{b}	0.261	0.011	0.020	0.080
Spirochaetaceae	1.291	0.777	0.770	0.860	0.184	1.112^{a}	0.736^{b}	0.120	0.185	0.030	0.234
Synergistaceae	1.133^{a}	3.287^{b}	3.288^{b}	2.397^{ab}	0.454	2.280	2.772	0.269	0.012	0.116	0.461
Acidaminococcaceae	1.612	2.148	1.860	1.575	0.173	1.907	1.691	0.114	0.112	0.154	0.081
Fibrobacteraceae	1.642	1.792	1.441	1.186	0.269	2.151^{a}	0.789^{b}	0.151	0.439	< 0.001	0.061
Anaeroplasmataceae	0.504	0.526	0.244	0.481	0.145	0.481	0.396	0.075	0.505	0.312	0.251
Selenomonadaceae	1.525	1.747	1.540	1.164	0.300	1.638	1.350	0.201	0.594	0.283	0.731
Hungateiclostridiaceae	0.487^{t}	0.372	0.229^{t}	0.382	0.066	0.473	0.263	0.037	0.089	< 0.001	0.910
Eubacteriaceae	0.514^{a}	0.332^{b}	0.307^{b}	0.333^{b}	0.052	0.376	0.367	0.030	0.046	0.761	0.111
Desulfovibrionaceae	0.210^{a}	0.780^{b}	0.610^{b}	0.610^{b}	0.114	0.574	0.531	0.067	0.018	0.566	0.887
Oscillospiraceae	0.531	0.893	0.795	0.990	0.171	0.682	0.923	0.101	0.296	0.083	0.367
Clostridiales Family XIII. Incertae Sedis	0.278^{a}	0.181^{b}	0.155^{b}	0.165^{b}	0.025	0.165^{a}	0.226^{b}	0.014	0.011	0.001	0.914
Bacteroidaceae	0.168^{a}	0.126^{b}	0.122^{b}	0.130^{ab}	0.014	0.136	0.137	0.008	0.050	0.853	0.253
Elusimicrobiaceae	0.201	0.076^{t}	0.190	0.239^{t}	0.044	0.116^{a}	0.237b	0.025	0.090	0.001	0.063
Endomicrobiaceae	0.186^{a}	0.072^{b}	0.081^{b}	0.088^{b}	0.023	0.082	0.131	0.018	0.009	0.128	0.589
Oxalobacteraceae	0.017^{a}	0.075^{b}	0.035^{ab}	0.004^{a}	0.016	0.040	0.026	0.009	0.031	0.094	0.739
Clostridiaceae	0.152	0.201	0.105	0.168	0.044	0.154	0.159	0.035	0.502	0.939	0.814
Victivallaceae	0.111	0.151	0.093	0.116	0.046	0.129	0.106	0.024	0.840	0.554	0.661
Methanobacteriaceae	0.104	0.128^{t}	0.046^{t}	0.055^{t}	0.024	0.075	0.091	0.016	0.077	0.455	0.967
Rhodospirillaceae	0.028	0.037	0.031	0.020	0.008	0.025	0.033	0.005	0.602	0.230	0.476
Christensenellaceae	0.229^{t}	0.051^{t}	0.086	0.150	0.048	0.130	0.128	0.028	0.083	0.962	0.758
Anaerolineaceae	0.063^{a}	0.179^{b}	0.141^{b}	0.222^{b}	0.028	0.168	0.134	0.020	0.007	0.297	0.938
Erysipelotrichaceae	0.068^{a}	0.020^{b}	0.015^{b}	0.014^{b}	0.008	0.029	0.030	0.004	< 0.001	0.827	0.651
Fusobacteriaceae	0.024^{t}	0.014	0.012	0.006^{t}	0.005	0.016	0.012	0.003	0.094	0.468	0.919
Oligosphaeraceae	0.052	0.028	0.029	0.057	0.010	0.035	0.047	0.007	0.116	0.201	0.586
Porphyromonadaceae	0.017^{t}	0.009	0.008	0.007	0.003	0.011	0.009	0.002	0.058	0.558	0.647
Atopobiaceae	0.059^{a}	0.019^{b}	0.016^{b}	0.013^{b}	0.006	0.025	0.029	0.004	< 0.001	0.215	0.332
Eggerthellaceae	0.024^{a}	0.014^{b}	0.011^{b}	0.008^{b}	0.002	0.013	0.015	0.001	0.001	0.259	0.463
Moraxellaceae	0.050	0.023	0.012	0.004	0.016	0.024	0.020	0.009	0.240	0.755	0.659
Pasteurellaceae	0.043	0.050	0.022	0.015	0.012	0.026	0.039	0.007	0.158	0.227	0.906
Bifidobacteriaceae	0.028	0.045	0.040	0.022	0.013	0.025^{a}	0.043b	0.007	0.604	0.025	0.344

Means with different superscript (a, b, c, d) between dietary treatments and (A, B) between sampling time differ significantly ($p \leq 0.05$) while, t; trend refers to $p < 0.10$. CON = control concentrate without microalgae; ALG20 = control concentrate with 20 g/Kg *Schizochytrium* sp.; ALG40 = control concentrate with 40 g/Kg *Schizochytrium* sp.; ALG60 = control concentrate with 60 g/Kg *Schizochytrium* sp. ‡ Effect: The dietary treatment (T), time (S), and the interaction between dietary treatment x time (TxS) effects were analyzed by ANOVA using a general linear model (GLM) for repeated measures and Post hoc analysis was performed when appropriate using Tukey multiple range test. †SEM: Standard error of the mean.

Table S5. Relative abundance of most abundant bacteria genera (> 0.06 %) and methanogenic archaea in the liquid of rumen of the four dietary treatments (CON, ALG20, ALG40, and ALG60) on two sampling points (20th and 40th experimental day).

Genera	Treatment (T)					Sampling time (S)			Effect ‡		
	CON	ALG20	ALG40	ALG60	SEM†	20	40	SEM†	T	S	T x S
Prevotella	18.485^{t}	21.607	22.245^{t}	18.666	1.370	19.601	20.901	0.897	0.099	0.271	0.859
Treponema	1.289	0.774	0.769	0.857	0.184	1.110^{a}	0.735^{b}	0.121	0.186	0.030	0.224
Ruminococcus	2.816^{a}	1.443^{b}	1.059^{b}	1.609^{b}	0.377	2.061^{a}	1.403^{b}	0.214	0.026	0.008	0.357
Fretibacterium	1.044^{a}	3.179^{b}	3.189^{b}	2.304^{ab}	0.451	2.196	2.663	0.262	0.012	0.133	0.019
Anaerocolumna	0.226	0.058	0.058	0.104	0.081	0.173	0.050	0.032	0.433	0.117	0.854
Ruminobacter	0.837^{a}	1.144^{ab}	2.061^{b}	0.677^{a}	0.344	1.292	1.067	0.231	0.049	0.498	0.181
Succiniclasticum	1.611	2.145	1.857	1.573	0.173	1.905	1.688	0.114	0.113	0.152	0.466
Succinivibrio	0.984	2.355	2.499	1.920	0.547	2.341	1.539	0.354	0.237	0.151	0.947
Fibrobacter	1.642	1.792	1.441	1.186	0.269	2.151^{a}	0.879^{b}	0.150	0.439	< 0.001	0.061
Anaeroplasma	0.485	0.492	0.210	0.453	0.144	0.462	0.358	0.072	0.476	0.208	0.219
Butyrivibrio	0.821	0.936^{t}	0.459^{t}	0.802	0.134	0.695	0.813	0.076	0.100	0.121	0.907
Flintibacter	0.908^{a}	0.137^{b}	0.130^{b}	0.152^{b}	0.115	0.373	0.290	0.065	< 0.001	0.188	0.067
Intestinimonas	1.143^{a}	0.348^{b}	0.129^{b}	0.240^{b}	0.090	0.472	0.458	0.068	< 0.001	0.873	0.785
Selenomonas	1.074	0.944	0.920	0.855	0.208	1.036	0.860	0.129	0.898	0.399	0.616
Eubacterium	0.512^{a}	0.332^{b}	0.307^{b}	0.331^{b}	0.052	0.375	0.365	0.030	0.046	0.742	0.112
Lachnoclostridium	0.499^{ab}	0.562^{a}	0.349^{b}	0.407^{b}	0.052	0.427	0.481	0.035	0.048	0.263	0.852
Acetatifactor	0.072	0.060	0.052	0.061	0.021	0.071	0.051	0.012	0.920	0.331	0.844
Saccharofermentans	0.326	0.295	0.175	0.286	0.055	0.372^{a}	0.169^{b}	0.032	0.272	< 0.001	0.736
Sporobacter	0.506^{a}	0.197^{b}	0.106^{b}	0.116^{b}	0.069	0.238	0.224	0.040	0.003	0.771	0.640
Desulfovibrio	0.199^{a}	0.772^{b}	0.600^{b}	0.595^{b}	0.115	0.559	0.524	0.069	0.019	0.634	0.857
Kineothrix	0.239	0.315	0.370	0.389	0.071	0.356	0.301	0.051	0.468	0.520	0.723
Pseudobutyrivibrio	0.370	0.361	0.248	0.269	0.050	0.284	0.340	0.029	0.241	0.087	0.521
Succinimonas	0.106	0.195	0.206	0.151	0.080	0.246^{a}	0.083^{b}	0.050	0.808	0.048	0.764
Ethanoligenens	0.126	0.081	0.136	0.112	0.050	0.142	0.085	0.027	0.872	0.106	0.344
Oscillibacter	0.531	0.893	0.795	0.990	0.171	0.682	0.923	0.104	0.293	0.083	0.367
Paraprevotella	0.099	0.027	0.028	0.049	0.029	0.046	0.056	0.017	0.299	0.607	0.595
Neglecta	0.064^{a}	0.007^{b}	0.004^{b}	0.004^{b}	0.012	0.031^{a}	0.008^{b}	0.007	0.007	0.034	0.064
Blautia	0.302^{a}	0.214^{b}	0.156^{b}	0.169^{b}	0.027	0.191	0.230	0.018	0.007	0.108	0.674
Anaerovibrio	0.343^{a}	0.703^{b}	0.517^{ab}	0.233^{a}	0.114	0.491	0.408	0.062	0.049	0.269	0.968
Oribacterium	0.182	0.225	0.129	0.165	0.037	0.158	0.192	0.030	0.357	0.489	0.379
Ruminococcaceae;null	0.194	0.122	0.143	0.268	0.051	0.115^{a}	0.248^{b}	0.031	0.225	0.020	0.278
Elusimicrobium	0.201	0.076^{t}	0.190	0.239^{t}	0.044	0.113^{a}	0.237^{b}	0.025	0.090	0.001	0.063
Bacteroides	0.133	0.112	0.118	0.125	0.012	0.124	0.120	0.007	0.651	0.609	0.354
Anaerosporobacter	0.061	0.061	0.076	0.052	0.016	0.081	0.044	0.012	0.779	0.069	0.649
Endomicrobium	0.186^{a}	0.072^{b}	0.081^{b}	0.088^{b}	0.023	0.082	0.131	0.018	0.009	0.128	0.111
Aminipila	0.142	0.099	0.093	0.096	0.018	0.089^{a}	0.126^{b}	0.010	0.219	0.015	0.857
Herbinix	0.117	0.087	0.349	0.068	0.077	0.162	0.148	0.052	0.069	0.854	0.547
Stomatobaculum	0.211^{a}	0.147^{b}	0.131^{b}	0.069^{c}	0.021	0.124	0.155	0.016	0.002	0.215	0.679
Hungateiclostridium	0.093^{a}	0.033^{b}	0.033^{b}	0.065^{ab}	0.014	0.062	0.050	0.009	0.018	0.358	0.438
Victivallis	0.111	0.151	0.093	0.116	0.046	0.129	0.106	0.023	0.840	0.554	0.542
Methanobrevibacter	0.102^{t}	0.126^{t}	0.045^{t}	0.054	0.024	0.074	0.089	0.016	0.083	0.472	0.973
Tyzzerella	0.097	0.139	0.108	0.165	0.024	0.143	0.111	0.019	0.225	0.271	0.841
Fusicatenibacter	0.060	0.087	0.060	0.049	0.015	0.064	0.065	0.012	0.352	0.512	0.966
Methanosphaera	0.002	0.002	0.001	0.001	< 0.001	0.001	0.002	< 0.001	0.635	0.416	0.875
Methanomicrobium	0.002^{t}	0.002^{t}	0.06	0.021^{t}	0.005	0.011	0.004	0.003	0.074	0.214	0.323
Methanimicrococcus	nd	nd	0.001	0.002	< 0.001	0.001	0.001	< 0.001	0.016	0.366	0.232
Methanomassiliicoccus	0.001	nd	0.001	nd	< 0.001	0.001	0.001	< 0.001	0.805	0.725	0.343

Means with different superscript (a, b, c, d) between dietary treatments and (A, B) between sampling time differ significantly ($p \leq 0.05$) while, t; trend refers to $p < 0.10$. CON = control concentrate without microalgae; ALG20 = control concentrate with 20 g/Kg *Schizochytrium* sp.; ALG40 = control concentrate with 40 g/Kg *Schizochytrium* sp.; ALG60 = control concentrate with 60 g/ Kg Schizochytrium sp. ‡ Effect: The dietary treatment (T), time (S), and the interaction between dietary treatment x time (TxS) effects were

analyzed by ANOVA using a general linear model (GLM) for repeated measures and Post hoc analysis was performed when appropriate using Tukey multiple range test. †SEM: Standard error of the mean. Denote the yellow highlighted genera were used in discriminant analyses (Figure 1).

Table S6. Relative abundance of most abundant bacteria species and methanogenic archaea in the liquid of rumen of the four dietary treatments (CON, ALG20, ALG40, and ALG60) on two sampling points (20th and 40th experimental day).

Species	Treatment (T)					Sampling time (S)			Effect ‡		
	CON	ALG20	ALG40	ALG60	SEM†	20	40	SEM†	T	S	T x S
Prevotella ruminicola	9.789	11.299	11.560	9.244	1.162	10.366	10.580	0.700	0.440	0.802	0.801
Fretibacterium fastidiosum	1.044^{a}	3.179^{b}	3.189^{b}	2.304^{b}	0.451	2.196	2.663	0.268	0.012	0.133	0.019
Prevotella brevis	1.339^{t}	1.526^{t}	2.216	2.589^{t}	0.374	2.006	1.829	0.202	0.097	0.575	0.343
Prevotella oralis	0.959	1.799	1.643	1.517	0.258	1.422	1.536	0.166	0.153	0.614	0.548
Ruminobacter amylophilus	0.837^{a}	1.144^{ab}	2.061^{b}	0.677^{a}	0.344	1.292	1.067	0.231	0.050	0.498	0.908
Succiniclasticum ruminis	1.611^{t}	2.145^{t}	1.857	1.573^{t}	0.173	1.905	1.688	0.113	0.098	0.152	0.710
Succinivibrio dextrinosolvens	0.984^{a}	2.355^{b}	2.499^{b}	1.920^{ab}	0.547	2.341	1.539	0.358	0.042	0.151	0.947
Fibrobacter succinogenes	1.620	1.692	1.432	1.186	0.270	2.096^{a}	0.869^{b}	0.150	0.566	< 0.001	0.126
Treponema bryantii	0.551^{a}	0.279^{b}	0.187^{b}	0.292^{b}	0.045	0.368^{a}	0.283s	0.025	< 0.001	0.047	0.075
Ruminococcus bromii	1.837^{a}	0.787^{b}	0.574^{b}	0.734^{b}	0.304	1.240^{a}	0.726^{b}	0.172	0.037	0.020	0.751
Prevotella corporis	0.516	0.367	0.437	0.502	0.266	0.607	0.303	0.155	0.977	0.186	0.433
Treponema saccharophilum	0.312	0.293	0.360	0.249	0.115	0.400	0.207	0.078	0.921	0.077	0.403
Anaeroplasma abactoclasticum	0.199	0.329	0.119	0.384	0.112	0.315	0.200	0.058	0.356	0.120	0.337
Butyrivibrio proteoclasticus	0.474	0.505	0.212	0.498	0.142	0.365	0.483	0.078	0.446	0.103	0.722
Prevotella copri	1.861	1.307	1.779	1.657	0.403	1.468	1.834	0.241	0.778	0.163	0.729
Flintibacter butyricus	0.908^{a}	0.137^{b}	0.130^{b}	0.152^{b}	0.115	0.373	0.290	0.065	< 0.001	0.188	0.067
Intestinimonas butyriciproducens	1.143^{a}	0.348^{b}	0.129^{b}	0.240^{b}	0.090	0.472	0.458	0.066	< 0.001	0.873	0.785
Prevotella shahii	0.805^{ab}	1.573^{a}	0.464^{b}	0.220^{b}	0.297	0.873	0.658	0.167	0.028	0.269	0.069
Acetatifactor muris	0.072	0.060	0.052	0.061	0.021	0.071	0.051	0.012	0.920	0.331	0.844
Saccharofermentans acetigenes	0.326	0.295	0.175	0.286	0.055	0.372^{a}	0.169^{b}	0.033	0.275	< 0.001	0.736
Sporobacter termitidis	0.506^{a}	0.197^{b}	0.106^{b}	0.116^{b}	0.069	0.238	0.224	0.041	0.003	0.771	0.640
Kineothrix alysoides	0.239	0.315	0.370	0.389	0.071	0.356	0.301	0.050	0.468	0.520	0.723
Pseudobutyrivibrio ruminis	0.368	0.360	0.247	0.268	0.050	0.283	0.339	0.033	0.240	0.087	0.516
Succinimonas amylolytica	0.106	0.195	0.206	0.151	0.080	0.246^{a}	0.083^{b}	0.050	0.808	0.048	0.764
Ethanoligenens harbinense	0.126	0.081	0.136	0.112	0.050	0.142	0.085	0.027	0.872	0.106	0.344
Paraprevotella clara	0.099	0.026	0.028	0.049	0.029	0.045	0.055	0.018	0.296	0.590	0.597
Treponema ruminis	0.321	0.165	0.180	0.260	0.074	0.284	0.181	0.049	0.420	0.150	0.675
Desulfovibrio desulfuricans	0.085^{a}	0.543^{b}	0.452^{b}	0.324^{ab}	0.111	0.379	0.323	0.063	0.049	0.407	0.606
Eubacterium coprostanoligenes	0.260^{a}	0.162^{a}	0.161^{a}	0.146^{a}	0.032	0.188	0.177	0.020	0.078	0.624	0.190
Ruminococcus flavefaciens	0.271^{a}	0.158^{ab}	0.097^{b}	0.268^{a}	0.040	0.190	0.208	0.023	0.016	0.494	0.463
Anaerovibrio lipolyticus	0.343^{a}	0.703^{b}	0.517^{ab}	0.233^{a}	0.114	0.491	0.408	0.062	0.049	0.269	0.968
Ruminococcus gauvreauii	0.179^{t}	0.105	0.069^{t}	0.110	0.027	0.151^{a}	0.080^{b}	0.019	0.070	0.017	0.162
Oribacterium asaccharolyticum	0.146	0.164	0.108	0.149	0.036	0.127	0.157	0.030	0.729	0.531	0.288
Oscillibacter valericigenes	0.154	0.483	0.500	0.699	0.158	0.437	0.482	0.090	0.178	0.683	0.536
Elusimicrobium minutum	0.201	0.076^{t}	0.190	0.239^{t}	0.044	0.116^{a}	0.237b	0.026	0.090	0.001	0.063
Selenomonas ruminantium	0.586	0.292	0.251	0.204	0.146	0.413	0.283	0.091	0.283	0.302	0.827
Prevotella micans	0.278	0.467	0.267	0.069	0.131	0.227	0.314	0.101	0.241	0.589	0.851
Ruminococcus albus	0.243^{t}	0.171	0.131^{t}	0.195	0.037	0.204^{a}	0.167b	0.020	0.099	0.036	0.600
Anaerosporobacter mobilis	0.061	0.061	0.076	0.052	0.016	0.081	0.044	0.012	0.779	0.069	0.614
Prevotella marshii	0.043^{t}	0.133^{t}	0.081	0.072	0.026	0.104	0.061	0.020	0.100	0.206	0.908
Eubacterium siraeum	0.119	0.073	0.086	0.213	0.047	0.070^{a}	0.176b	0.026	0.187	0.039	0.310
Clostridium clostridioforme	0.093	0.157	0.113	0.058	0.030	0.078	0.138	0.022	0.183	0.109	0.238
Endomicrobium proavitum	0.186^{a}	0.072^{b}	0.081^{b}	0.088^{b}	0.023	0.082	0.131	0.017	0.009	0.128	0.111
Prevotella amnii	0.032	0.177	0.096	0.404	0.119	0.199	0.155	0.087	0.176	0.772	0.578
Aminipila butyrica	0.142	0.099	0.093	0.096	0.018	0.089^{a}	0.126b	0.009	0.213	0.015	0.857
Prevotella oulorum	0.216	0.163	0.530	0.210	0.213	0.211	0.349	0.120	0.608	0.125	0.572

Ruminococcus champanellensis	0.210	0.173	0.135	0.257	0.048	0.217	0.171	0.026	0.356	0.054	0.501
Prevotella buccalis	0.200	0.249	0.150	0.188	0.053	0.176	0.218	0.036	0.636	0.353	0.606
Herbinix luporum	0.117^{t}	0.086	0.343^{t}	0.067^{t}	0.077	0.162	0.147	0.052	0.068	0.854	0.543
Oscillibacter ruminantium	0.376	0.410	0.295	0.291	0.123	0.245^{a}	0.441b	0.074	0.871	0.009	0.688
Prevotella oris	0.267	0.800	0.410	0.161	0.226	0.243	0.575	0.120	0.246	0.122	0.252
Stomatobaculum longum	0.211^{a}	0.147b	0.131b	0.069c	0.021	0.124	0.155	0.016	0.002	0.215	0.679
Victivallis vadensis	0.111	0.151	0.093	0.116	0.046	0.129	0.106	0.022	0.840	0.554	0.661
Desulfovibrio intestinalis	0.041^{a}	0.153^{b}	0.099^{ab}	0.215^{b}	0.039	0.120	0.134	0.026	0.036	0.683	0.840
Prevotella paludivivens	0.086	0.074	0.092	0.048	0.017	0.063	0.087	0.010	0.306	0.064	0.030
Prevotella stercorea	0.169	0.293	0.390	0.433	0.126	0.437	0.206	0.075	0.480	0.109	0.340
Selenomonas lacticifex	0.015	0.028	0.028	0.038	0.010	0.032	0.023	0.005	0.440	0.196	0.608
Blautia luti	0.121^{a}	0.059^{b}	0.087^{ab}	0.052^{b}	0.016	0.079	0.078	0.012	0.034	0.963	0.089
Clostridium asparagiforme	0.104	0.156	0.046	0.076	0.033	0.095	0.096	0.020	0.144	0.973	0.714
Fusicatenibacter saccharivorans	0.060	0.087	0.060	0.049	0.015	0.064	0.065	0.013	0.352	0.966	0.512
Butyrivibrio hungatei	0.294	0.375	0.222	0.289	0.056	0.309	0.281	0.047	0.322	0.719	0.718
Selenomonas flueggei	0.097	0.176	0.148	0.132	0.056	0.179	0.098	0.037	0.788	0.092	0.541
Anaerotaenia torta	0.080	0.051	0.035	0.058	0.017	0.070^{a}	0.042b	0.009	0.356	0.046	0.294
Murimonas intestini	0.078	0.056	0.044	0.053	0.010	0.054	0.061	0.007	0.123	0.454	0.069
Prevotella colorans	0.133	0.071	0.084	0.066	0.042	0.096	0.081	0.024	0.676	0.622	0.809
Methanobrevibacter thaueri	0.027	0.042	0.012	0.016	0.013	0.012	0.037	0.008	0.377	0.078	0.079
Butyrivibrio fibrisolvens	0.048	0.055	0.018	0.014	0.015	0.021	0.047	0.009	0.161	0.065	0.869
Faecalicatena orotica	0.077	0.020	0.012	0.009	0.007	0.028	0.031	0.005	< 0.001	0.759	0.953
Hungateiclostridium alkalicellulosi	0.048^{a}	0.004^{b}	0.009^{b}	0.021^{b}	0.009	0.019	0.022	0.006	0.016	0.784	0.981
Prevotella dentalis	0.123	0.073	0.437	0.074	0.152	0.096	0.257	0.075	0.310	0.315	0.180
Ruminococcus faecis	0.031	0.026	0.023	0.021	0.005	0.033^{a}	0.018b	0.003	0.569	0.003	0.878
Anaerocolumna cellulosilytica	0.067	0.049	0.050	0.056	0.015	0.070	0.041	0.008	0.812	0.066	0.955
Ihubacter massiliensis	0.060^{a}	0.041b	0.030b	0.032b	0.007	0.034^{a}	0.048b	0.004	0.018	0.025	0.757
Eubacterium ruminantium	0.075	0.094	0.088	0.103	0.025	0.088	0.092	0.015	0.887	0.797	0.078
Prevotella melaninogenica	0.235	0.337	0.498	0.236	0.201	0.316	0.337	0.121	0.769	0.907	0.061
Coprococcus eutactus	0.055	0.034	0.031	0.042	0.009	0.044	0.037	0.006	0.288	0.463	0.249
Prevotella bryantii	0.157	0.046	0.028	0.033	0.027	0.049	0.083	0.017	0.010	0.273	0.249
Ruminococcus lactaris	0.032^{a}	0.010^{b}	0.006^{b}	0.006^{b}	0.003	0.013	0.014	0.003	< 0.001	0.770	0.981
Prevotella loescheii	0,331	0,034	0,186	0,140	0,114	0,102	0,243	0,080	0,355	0,317	0,883
Tyzzerella nexilis	0,081	0,078	0,035	0,090	0,017	0,069	0,073	0,012	0,141	0,845	0,885
Eubacterium hallii	0,055	0,044	0,029	0,040	0,011	0,042	0,042	0,006	0,462	0,965	0,365
Eubacterium oxidoreducens	0,062^{a}	0,008^{b}	0,007^{b}	0,012^{b}	0,012	0.027	0.017	0.007	0,013	0.183	0.275
Selenomonas dianae	0,046	0,079	0,089	0,092	0,016	0.070	0.082	0.012	0,183	0.544	0.043
Selenomonas noxia	0,181	0,080	0,123	0,175	0,058	0,162	0,118	0,040	0,580	0,495	0,736
Clostridium colinum	0,016	0,062	0,072	0,076	0,016	0,074	0,039	0,011	0,062	0,051	0,381
Clostridium methylpentosum	0,050	0,034	0,040	0,013	0,017	0,020	0,049	0,011	0,505	0,118	0,777
Lachnoclostridium pacaense	0,032	0,041	0,041	0,065	0,009	0,050	0,039	0,007	0,113	0,353	0,458
Hungateiclostridium straminisolvens	0,015	0,010	0,004	0,007	0,004	0,010	0,008	0,002	0,296	0,179	0,379
Selenomonas sputigena	0,097^{t}	0,247^{t}	0,247^{t}	0,152	0,046	0.131^{a}	0.241b	0.034	0,092	0.045	0.541
Abyssivirga alkaniphila	0,048^{a}	0,024^{b}	0,015^{b}	0,012^{b}	0,004	0.026	0.023	0.003	< 0.001	0.585	0.581
Clostridium scindens	0,042^{a}	0,017^{b}	0,009^{b}	0,010^{b}	0,005	0.020	0.019	0.003	0.001	0.588	0.688
Bacteroides stercoris	0,017	0,014	0,015	0,012	0,002	0,015	0,014	0,001	0,473	0,322	0,378
Bacteroides vulgatus	0,014	0,011	0,016	0,015	0,003	0,013	0,015	0,002	0,602	0,401	0,486
Pseudoflavonifractor phocaeensis	0,024	0,006	0,009	0,032	0,009	0,014	0,021	0,005	0,192	0,426	0,557
Prevotella denticola	0,124	0,006	0,012	0,008	0,055	0,036	0,039	0,027	0,376	0,109	0,244
Prevotella saccharolytica	0,019	0,060	0,048	0,027	0,017	0,037	0,040	0,009	0,321	0,863	0,597
Selenomonas bovis	0,042	0,031	0,025	0,028	0,013	0,034	0,030	0,009	0,774	0,769	0,571
Solobacterium moorei	0,037^{a}	0,010b	0,006b	0,006b	0,006	0.016	0.013	0.004	0,004	0.766	0.507
Faecalibacterium prausnitzii	0,025	0,014	0,019	0,016	0,004	0,020	0,017	0,003	0,279	0,326	0,100
Prevotella dentasini	0,023	0,005	0,012	0,005	0,006	0,014	0,008	0,003	0,178	0,339	0,737
Clostridium lavalense	0,018	0,022	0,016	0,054	0,014	0,028	0,026	0,007	0,204	0,828	0,740

Eubacterium rectale	0,015	0,012	0,016	0,020	0,005	0,009^{a}	0,022b	0,002	0,672	0,006	0.637
Lachnoanaerobaculum orale	0,006^{a}	0,014	0,024	0,064b	0,014	0.035	0.019	0.010	0,051	0.326	0.890
Mitsuokella multacida	0,066	0,078	0,073	0,060	0,014	0,081	0,058	0,008	0,846	0,132	0,876
Clostridium aminophilum	0,048	0,066	0,043	0,060	0,009	0,044^{a}	0,065b	0.006	0,312	0.007	0.936
Ruminococcus callidus	0,014	0,011	0,021	0,018	0,008	0,013	0,019	0,004	0,811	0,165	0,933
Prevotella maculosa	0,058	0,166	0,326	0,140	0,152	0,082	0,263	0,081	0,657	0,236	0,458
Ruminiclostridium cellulolyticum	0,021	0,016	0,007	0,009	0,005	0,012	0,015	0,004	0,176	0,727	0,894
Clostridium leptum	0,018	0,005	0,006	0,028	0,012	0,012	0,017	0,006	0,453	0,495	0,304
Clostridium saudiense	0,003	0,003	0,003	0,003	0,001	0,004^{a}	0,001b	0,001	0,976	0,001	0.721
Collinsella aerofaciens	0,006	0,002	0,003	0,004	0,001	0,005	0,003	0,001	0,167	0,407	0,040
Clostridium populeti	0,021	0,025	0,014	0,013	0,007	0,018	0,018	0,004	0,566	0,959	0,326
Clostridium xylanolyticum	0,027	0,026	0,007	0,022	0,008	0,030^{a}	0,011b	0,004	0,330	0,001	0.104
Methanobrevibacter olleyae	0,006α	0,001b	0,000b	0,000b	<0,001	0.002	0.002	<0,001	<0,001	0.295	0.084
Methanobrevibacter millerae	0,066	0,075	0,027	0,033	0,021	0,056	0,044	0,013	0,336	0,481	0,053
Methanomicrobium mobile	0,002^{a}	0,002^{a}	0,006^{a}b	0,021b	0,005	0.011	0.004	0.003	0,074	0.214	0.323
Ruminococcus gnavus	0,005	0,007	0,004	0,011	0,002	0,008	0,006	0,001	0,160	0,377	0,175
Ruminococcus torques	0,003	0,002	0,002	0,001	0,001	0,002	0,002	0,001	0,666	0,703	0,215
Streptococcus pharyngis	0,003	0,001	0,001	<0,001	0,001	0,002	0,001	<0,001	0,127	0,297	0,288
Methanobrevibacter boviskoreani	0,003	0,004	0,004	0,003	0,002	0,002	0,005	0,001	0,957	0,216	0,890
Methanimicrococcus blatticola	<0,001^{a}	<0,001^{a}	0,001^{a}b	0,002b	<0,001	0.001	0.001	<0,001	0,016	0.366	0.232
Methanobrevibacter ruminantium	-	-	-	-	-	-	-	-	-	-	-
Methanobrevibacter smithii	-	-	-	-	-	-	-	-	-	-	-
Methanomassiliicoccus luminyensis	0,001	0,000	0,001	0,000	<0,001	0,000	0,001	<0,001	0,805	0,725	0,343
Methanobrevibacter boviskoreani	0,003	0,004	0,004	0,003	0,002	0,002	0,005	0,001	0,957	0,216	0,890
Methanosphaera stadtmanae	0,002	0,002	0,001	0,001	0,001	0,001	0,002	<0,001	0,635	0,416	0,762

Means with different superscript (a, b, c, d) between dietary treatments and (A, B) between sampling time differ significantly ($p \leq 0.05$) while, t; trend refers to $p < 0.10$. CON = control concentrate without microalgae; ALG20 = control concentrate with 20 g/Kg *Schizochytrium* sp.; ALG40 = control concentrate with 40 g/Kg *Schizochytrium* sp.; ALG60 = control concentrate with 60 g/Kg *Schizochytrium* sp. ‡ Effect: The dietary treatment (T), time (S), and the interaction between dietary treatment x time (TxS) effects were analyzed by ANOVA using a general linear model (GLM) for repeated measures and Post hoc analysis was performed when appropriate using Tukey multiple range test. †SEM: Standard error of the mean Yellow highlighted; species used as proteolytic (Figure 4A,B). Blue highlighted; species used as amylolytic (Figure 5A,B). Green highlighted; species used as cellulolytic (Figure 6A,B). Grey highlighted; species used as other important bacteria (Figure 7). Purple highlighted; species used as methanogenic (Figure 8).

Table S7. Ingredients of concentrate (g/Kg), average feed consumption (g/goat/day), intake of nutrients and fatty acids (g/goat) of the four diets.

**Ingredients (g/Kg)**		**Concentrate**		
Maize grain		367		
Barley grain		200		
Wheat middlings		210		
Sunflower meal		80		
Soybean meal		100		
Calcium phosphate		15		
Calcium carbonate		5		
Salt		3		
Mineral and vitamin		20		
Daily feed consumption (g/goat)	**CON**	**ALG20**	**ALG40**	**ALG60**
Alfalfa hay	1000	1000	1000	1000
Concentrate	1000	1000	1000	**670***
Daily intake of nutrients (g/goat)				
Dry matter	1849	1854	1854	1547
Crude protein	260	261	261	219
Ether extract	56	70	76	66
Crude fiber	369	371	371	357
NDF	660	655	652	621

ADF	442	437	438	424
Fatty acids intake (g/goat/day)				
$C_{14:0}$	0.34	1.10	2.06	3.08
$C_{16:0}$	10.26	12.77	15.88	14.01
$C_{18:0}$	2.43	2.58	2.46	2.08
Cis-9 $C_{18:1}$	17.03	18.49	16.75	13.05
$C_{18:2\ n\text{-}6}$	21.58	24.23	23.03	18.57
$C_{18:3\ n\text{-}3}$	2.78	3.47	3.38	2.90
$C_{22:5\ n\text{-}6}$	-	1.65	3.34	3.28
$C_{22:6\ n\text{-}3}$	-	4.16	8.44	8.20

*denotes that concentrate were decreased in ALG60 by 33%. CON = control concentrate without microalgae *(Schizochytrium sp)*; ALG20 = CON with 20 g/Kg *Schizochytrium sp.*; ALG40 = CON with 40 g/Kg.; ALG60 = CON with 60 g/Kg *Schizochytrium sp.;* NDF = Neutral detergent fiber; ADF = Acid detergent fiber.

Table S8. Effect of supplementing microalgae *Schizochytrium sp* on pH, ammonia concentration, a-amylase, protease, cellulase, and xylanase activity in the liquid of rumen of the four dietary treatments (CON, ALG20, ALG40, and ALG60) on two sampling points (20th and 40th experimental day).

	**Treatment (T)**					**Sampling time (S)**			**Effect‡**		
	CON	ALG20	ALG40	ALG60	SEM†	20	40	SEM†	T	S	T * S
Protease activity (U/mL) [a]	0.106	0.142	0.144	0.137	0.019	0.114	0.150	0.015	0.456	0.234	0.991
a-Amylase activity (U/mL) [b]	2.738^{a}	2.784^{a}	3.483^{b}	2.822^{a}	0.207	2.837	3.077	0.148	0.048	0.313	0.641
Cellulose activity (U/mL/h) [c]	11.29^{a}	11.07^{ab}	10.28^{b}	9.76^{b}	0.450	9.95^{a}	11.25^{b}	0.287	0.050	0.008	0.248
Xylanase (U/mL/h) [d]	8.09	8.32	8.75	7.72	0.400	8.13	8.31	0.247	0.356	0.555	0.599
NH3-N mg/L	123.7^{a}	124.1^{a}	93.3^{b}	114.0^{a}	6.137	105.1A	122.5B	4.201	0.008	0.008	0.018
pH	7.30	7.36	7.12	7.24	0.057	7.24	7.27	0.044	0.120	0.703	0.868

Means with different superscript (a, b, c, d) between dietary treatments and (A, B) between sampling time differ significantly ($p \le 0.05$). CON = control concentrate without microalgae; ALG20 = control concentrate with 20 g/Kg *Schizochytrium* sp.; ALG40 = control concentrate with 40 g/Kg *Schizochytrium* sp.; ALG60 = control concentrate with 60 g/Kg *Schizochytrium* sp. ‡ Effect: The dietary treatment (T), time (S), and the interaction between dietary treatment x time (TxS) effects were analyzed by ANOVA using a general linear model (GLM) for repeated measures and Post hoc analysis was performed when appropriate using Tukey multiple range test. †SEM: Standard error of the mean a Units/mL= [(μmol of protein equivalent to ABS) x (total volume of reaction in mL) x dilution factor] / [(incubation time in minutes) x (volume of colorimetric determination in mL) x (volume of rumen sample in mL)]. b One unit of α-amylase activity will liberate 1.0 mg of maltose from starch in 10 min at pH 6.9 at 39 °C. c One unit of cellulase activity based on endo-cellulase (*A. niger*) clearance activity in 24h incubation at 50 °C. d One unit of xylanase activity based on endo-1-4-beta-Xylanase M1 (*T. viride*) clearance activity in 24h incubation at 37 °C.

Table S9. The mean individual fatty acids (FA) (% of total FA) of goats' rumen fluid of the four dietary treatments (CON, ALG20, ALG40, and ALG60) on two sampling points (20th and 40th experimental day).

	**Treatment (T)**					**Sampling time (S)**			**Effect ‡**		
	CON	ALG20	ALG40	ALG60	SEM†	20	40	SEM†	T	S	TxS
**$C_{14:0}$**	1.61^{a}	2.86^{b}	3.74^{c}	4.37^{d}	0.142	3.16	3.13	0.101	<0.001	0.857	0.367
**$C_{14:1}$**	1.06	1.51	1.11	1.24	0.117	1.21	1.24	0.087	0.056	0.732	0.002
**$C_{15:0}$**	1.06	1.36	0.92	0.96	0.100	0.99	1.16	0.060	0.022	0.093	0.019
**$C_{16:0}$**	26.64^{a}	29.36^{b}	25.27^{a}	25.22^{a}	0.545	25.26^{A}	27.98^{B}	0.345	<0.001	<0.001	0.008
**$C_{16:1}$**	0.209	0.512	0.324	0.612	0.113	0.305^{A}	0.524^{B}	0.069	0.082	0.021	0.398
**$C_{18:0}$**	44.27^{a}	6.62^{b}	3.67^{b}	3.39^{b}	1.483	14.75	14.23	0.821	<0.001	0.509	0.113
**trans $C_{18:1}$**	1.48^{a}	3.06^{b}	2.08^{a}	1.85^{a}	0.283	2.25	1.94	0.159	0.005	0.295	0.159
**trans-11 $C_{18:1}$**	4.80^{a}	27.47^{b}	25.84^{b}	23.14^{b}	1.212	21.96^{A}	18.66^{B}	0.702	<0.001	<0.001	<0.001
**trans-10 $C_{18:1}$**	0.00^{a}	2.18^{b}	1.64^{b}	1.59^{b}	0.138	1.50^{A}	1.21^{B}	0.098	<0.001	<0.001	0.241
**cis-9 $C_{18:1}$**	9.86^{a}	12.32^{b}	9.65^{a}	8.34^{a}	0.552	9.19^{A}	10.91^{B}	0.345	0.001	<0.001	0.241
**$C_{18:2\ n\text{-}6\ cis}$**	6.23^{a}	4.53^{ab}	3.77^{b}	3.18^{b}	0.509	4.07	4.79	0.321	0.003	0.104	0.262
**$C_{18:3\ n\text{-}3}$**	1.27	1.01	0.98	1.04	0.268	0.816	1.34	0.191	0.866	0.058	0.305
**$C_{18:2\ conjugated}$**	1.07	1.43	1.36	1.25	0.181	0.969^{A}	1.59^{B}	0.102	0.521	0.001	0.278
**$C_{22:5\ n\text{-}6}$**	0.00^{a}	2.32^{b}	6.81^{c}	8.33^{d}	0.248	4.64^{A}	4.09^{B}	0.191	<0.001	0.034	0.010
**$C_{22:6\ n\text{-}3}$**	0.00^{a}	3.08^{b}	12.72^{c}	15.32^{d}	0.547	8.70^{A}	6.86^{B}	0.359	<0.001	0.003	0.030

Means with different superscript (a, b, c, d) between dietary treatments and (A, B) between sampling time differ significantly ($p \le 0.05$). CON = control concentrate without microalgae; ALG20 = control concentrate with 20 g/Kg *Schizochytrium* sp.; ALG40 = control concentrate with 40 g/Kg *Schizochytrium* sp.; ALG60 = control concentrate with 60 g/Kg *Schizochytrium* sp. ‡ Effect: The dietary treatment (T), time (S), and the interaction between dietary treatment x time (TxS) effects were analyzed by ANOVA using a general linear model (GLM) for repeated measures and Post hoc analysis was performed when appropriate using Tukey multiple range test. †SEM: Standard error of the mean.

Table S10. The mean individual fatty acids (FA) (% of total FA), FA groups, SFA/UFA and ω6/ω3 ratios and Δ-9 desaturate indexes of milk from goats fed diets with different levels (g/kg concentrate) of microalgae Schizochytrium sp. (CON, ALG20, ALG40 and ALG60) on two sampling points (20th and 40th experimental day).

	**Treatment (T)**					**Sampling time (S)**			**Effect ‡**		
	CON	ALG20	ALG40	ALG60	SEM†	20	40	SEM†	T	S	TxS
$C_{4:0}$	2.26	2.43	2.44	2.15	0.131	2.09^{A}	2.54^{B}	0.072	0.401	0.001	0.008
$C_{6:0}$	2.73	2.77	2.89	2.80	0.120	2.66^{A}	2.93^{B}	0.073	0.807	0.005	0.374
$C_{8:0}$	3.31	3.28	3.61	3.72	0.180	3.40	3.57	0.112	0.273	0.232	0.344
$C_{10:0}$	10.77	10.54	11.43	12.19	0.700	10.73	11.73	0.419	0.39	0.061	0.371
$C_{12:0}$	4.49	4.16	4.75	5.07	0.400	4.35	4.88	0.244	0.452	0.071	0.572
$C_{14:0}$	9.92^{a}	9.99^{a}	12.79^{b}	11.28^{ab}	0.583	10.61	11.38	0.400	0.011	0.211	0.257
$C_{14:1}$	0.30^{a}	0.28^{ab}	0.24^{b}	0.30^{ab}	0.025	0.32^{A}	0.24^{B}	0.012	0.265	0.001	0.001
$C_{15:0}$	0.90^{ab}	0.97^{a}	0.78^{b}	0.86^{ab}	0.043	0.92^{A}	0.83^{B}	0.026	0.046	0.008	0.139
$C_{15:1}$	0.20^{a}	0.20^{ab}	0.17^{b}	0.19^{ab}	0.015	0.22^{A}	0.16^{B}	0.011	0.503	0.001	0.591
$C_{16:0}$	24.13^{a}	27.55^{b}	27.67^{b}	28.12^{b}	1.164	26.10^{A}	27.64^{B}	0.664	0.071	0.044	0.511
$C_{16:1}$	0.96^{a}	0.77^{ab}	0.71^{b}	0.66^{b}	0.081	0.68^{A}	0.87^{B}	0.046	0.077	0.003	0.314
$C_{17:0}$	0.61^{a}	0.37^{b}	0.28^{b}	0.28^{b}	0.066	0.39^{A}	0.39^{A}	0.040	0.006	0.973	0.723
$C_{18:0}$	9.66^{a}	2.97^{b}	1.85^{b}	1.85^{b}	0.660	4.33	3.84	0.350	< 0.001	0.170	0.653
trans $C_{18:1}$††	0.71^{a}	1.25^{b}	1.01^{ab}	0.95^{ab}	0.103	1.09^{B}	0.87^{A}	0.062	0.009	0.050	0.627
trans-10 $C_{18:1}$	0.61^{a}	0.96^{b}	0.82^{b}	0.82^{b}	0.064	0.83	0.77	0.038	0.005	0.191	0.227
trans-11 $C_{18:1}$	1.59^{a}	10.36^{b}	9.25^{b}	9.80^{b}	0.700	9.01^{A}	6.49^{B}	0.436	< 0.001	< 0.001	0.009
cis-9 $C_{18:1}$	21.86^{a}	11.57^{b}	8.93^{b}	8.11^{b}	1.280	12.65	12.58	0.734	< 0.001	0.942	0.437
$C_{18:2\ n\text{-}6\ t}$	0.29	0.37	0.34	0.28	0.031	0.29^{A}	0.35^{B}	0.019	0.171	0.017	0.389
$C_{18:2\ n\text{-}6\ c}$	2.84^{a}	2.79^{a}	1.93^{b}	1.60^{b}	0.165	2.21	2.37	0.095	< 0.001	0.124	0.170
$C_{18:3\ n\text{-}3}$	0.24	0.26	0.25	0.26	0.033	0.21^{A}	0.30^{B}	0.017	0.919	< 0.001	0.220
cis-9, trans-11 $C_{18:2}$	0.96^{a}	3.87^{b}	4.36^{b}	4.21^{b}	0.360	3.82^{A}	2.87^{B}	0.188	< 0.001	< 0.001	0.009
trans-10, cis-12 $C_{18:2}$	0.03^{a}	0.17^{b}	0.18^{b}	0.18^{b}	0.022	0.13	0.15	0.015	< 0.001	0.527	0.045
$C_{20:3\ n\text{-}3}$	0.23^{a}	0.33^{b}	0.52^{c}	0.55^{c}	0.030	0.41^{A}	0.40^{A}	0.017	< 0.001	0.338	0.490
$C_{22:5\ n\text{-}6}$	0.00^{a}	0.25^{b}	0.53^{c}	0.74^{d}	0.034	0.44^{A}	0.32^{B}	0.020	< 0.001	< 0.001	0.001
$C_{22:6\ n\text{-}3}$	0.00^{a}	0.70^{b}	1.26^{c}	1.55^{d}	0.075	0.96^{A}	0.80^{B}	0.043	< 0.001	0.003	0.001
**SCFA**	19.10	19.09	20.37	20.90	0.900	18.93^{A}	20.80^{B}	0.565	0.410	0.020	0.407
**MCFA**	39.46^{a}	42.69^{ab}	45.98^{b}	45.35^{b}	1.705	42.01^{A}	44.74^{B}	0.994	0.054	0.018	0.184
**LCFA**	10.39^{a}	3.70^{b}	2.60^{b}	2.75^{b}	0.705	5.20	4.52	0.380	< 0.001	0.116	0.729
**MUFA**	26.46^{a}	25.75^{ab}	21.64^{b}	21.58^{b}	1.445	25.33^{A}	22.38^{B}	0.926	0.050	0.031	0.292
**PUFA**	4.59^{a}	8.77^{b}	9.41^{b}	9.42^{b}	0.420	8.54^{A}	7.56^{B}	0.225	< 0.001	< 0.001	0.001
**SFA**	68.95	65.48	68.95	69.00	1.600	66.13^{A}	70.06^{B}	1.009	0.287	0.009	0.119
**UFA**	31.05	34.52	31.05	31.00	1.600	33.87^{A}	29.94^{B}	1.009	0.287	0.009	0.119
**SFA/UFA**	2.25	1.97	2.27	2.35	0.155	2.03^{A}	2.40^{B}	0.100	0.308	0.009	0.036
ω6	3.15^{ab}	3.44^{a}	2.84^{b}	2.70^{b}	0.175	3.02	3.04	0.105	0.035	0.852	0.072
ω3	0.46^{a}	1.29^{b}	2.03^{c}	2.41^{d}	0.090	1.60^{A}	1.50^{B}	0.053	< 0.001	0.044	0.000
ω6/ω3	7.3^{a}	2.72^{b}	1.42^{bc}	1.13^{c}	0,500	3.04	3.24	0.308	< 0.001	0.690	0.966
AI	2.24	2.18	2.74	2.68	0.190	2.23^{A}	2.68^{B}	0.124	0.111	0.011	0.029
TI	2.77	2.38	2.21	2.20	0.153	2.22^{A}	2.49^{B}	0.093	0.052	0.031	0.011
HPI	0.44	0.42	0.33	0.34	0.036	0.43	0.35	0.029	0.486	0.648	0.978
						**Δ^{-9} desaturase index**					
$C_{14:1}$ / $C_{14:0}$	0.031	0.030	0.019	0.029	0.003	0.032^{A}	0.022^{B}	0.002	0.100	0.001	0.036
$C_{16:1}$ / $C_{16:0}$	0.040^{a}	0.029^{b}	0.026^{b}	0.023^{b}	0.003	0.027^{A}	0.032^{B}	0.002	0.008	0.047	0.534
cis-9 $C_{18:1}$ / $C_{18:0}$	2.28^{a}	4.58^{b}	4.91^{b}	4.55^{b}	0.325	3.77^{A}	4.39^{B}	0.178	< 0.001	0.001	0.042
cis-9, trans-11 $C_{18:2}$ / *trans-11* $C_{18:1}$	0.61^{a}	0.38^{b}	0.44^{ab}	0.46^{b}	0.044	0.48	0.48	0.026	0.006	0.968	0.055

Means with different superscript (a, b, c, d) between dietary treatments and (A, B) between sampling time differ significantly ($p \leq 0.05$). CON = control concentrate without microalgae; ALG20 = control concentrate with 20 g/Kg *Schizochytrium* sp.; ALG40 = control concentrate with 40 g/Kg *Schizochytrium* sp.; ALG60 = control concentrate with 60 g/Kg *Schizochytrium* sp. ‡ Effect: The dietary treatment (T), time (S), and the interaction between dietary treatment x time (TxS) effects were analyzed by ANOVA using a general linear model (GLM) for repeated measures and Post hoc analysis was performed when appropriate using Tukey multiple range test.

†SEM: Standard error of the mean †† trans C18:1 proportion are not including the trans-11 C18:1 and trans-10 C18:1 content Short-Chain Saturated Fatty Acids (SCFA) = C6:0 + C8:0 + C10:0 + C11:0, Medium-Chain Saturated Fatty Acids (MCFA) = C12:0 + C13:0 + C14:0 + C15:0 + C16:0, Long-Chain Saturated Fatty Acids (LCFA) = C17:0 + C18:0 + C20:0, Mono-Unsaturated Fatty Acids (MUFA) = C14:1 + C15:1 + C16:1 + C17:1 + cis-9 C18:1 + trans-11 C18:1 + trans C18:1, Poly-Unsaturated Fatty Acids (PUFA) = cis-9, trans-11 C18:2 + C18:2n-6c + C18:2n-6t + C18:3n-3 + C18:3n-6 + C20:3n-3, Saturated Fatty Acids (SFA) = SCFA + MCFA + LCFA, Unsaturated Fatty Acids (UFA) = PUFA + MUFA, Saturated/Unsaturated (S/U) = (SCFA + MCFA + LCFA)/(PUFA + MUFA), Atherogenic index (AI) = (C12:0 + 4 x C14:0 + C16:0)/(PUFA + MUFA), Thrombogenic index (TI) = (C14:0 + C16:0 + C18:0)/(0.5 × MUFA) + (0.5 × n-6 PUFA) + (3 × ω3 PUFA) + (ω3 PUFA/ω6 PUFA), Health promoting index (HPI) = (ω6 PUFA + ω3 PUFA + MUF/(C12:0 + 4 × C14:0 + C16:0).

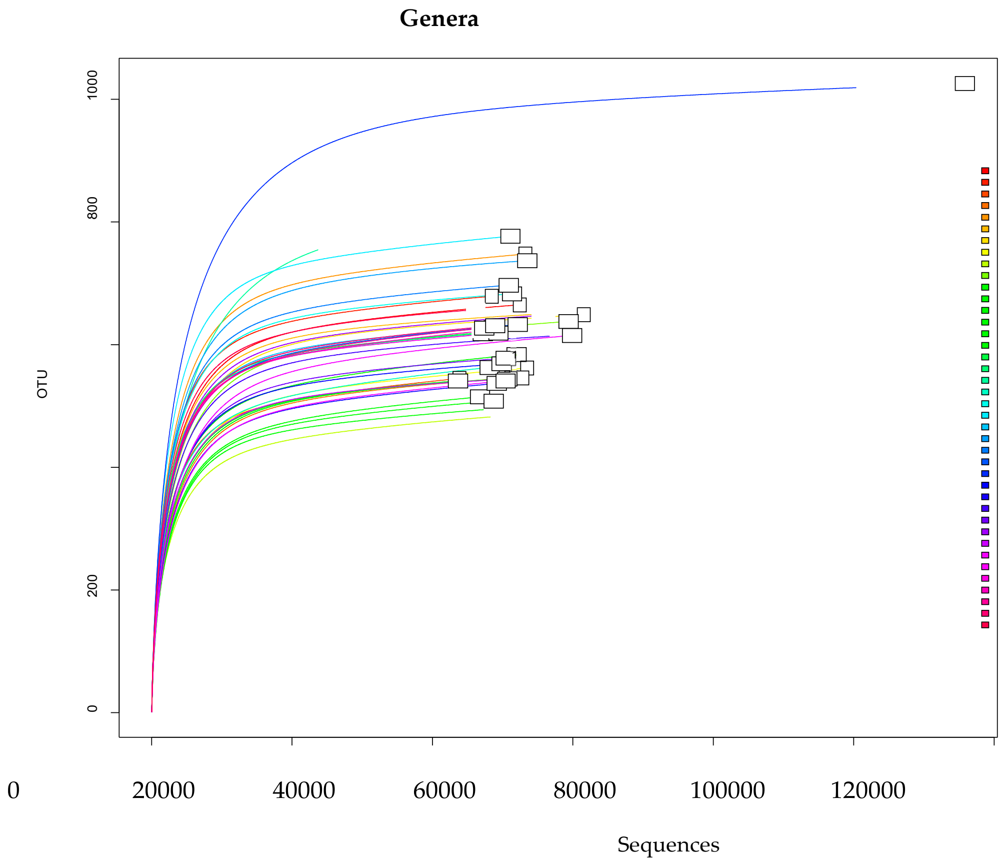

Figure S1. Rarefaction curves of bacterial population at genera taxonomic level for the 16S.

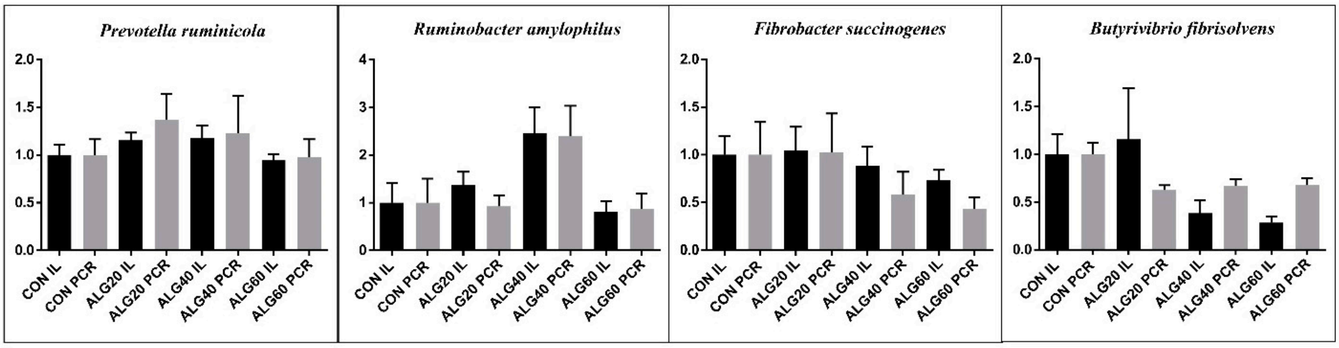

Figure S2. Validation of the 16s rRNA sequencing (IL) by qPCR (PCR) on the relative abundances of Prevotella ruminicola, *Ruminobacter amylophilus*, *Fibrobacter succinogenes*, and *Butyrivibrio fibrisolvens*. The microalgae treated groups (ALG20, ALG40, and ALG60) of each method (IL *vs.* PCR) have been expressed relatively as fold changes towards to the control (CON) group in order to be similarly visualized. Data are depicted as trends and no statistic has been applied.

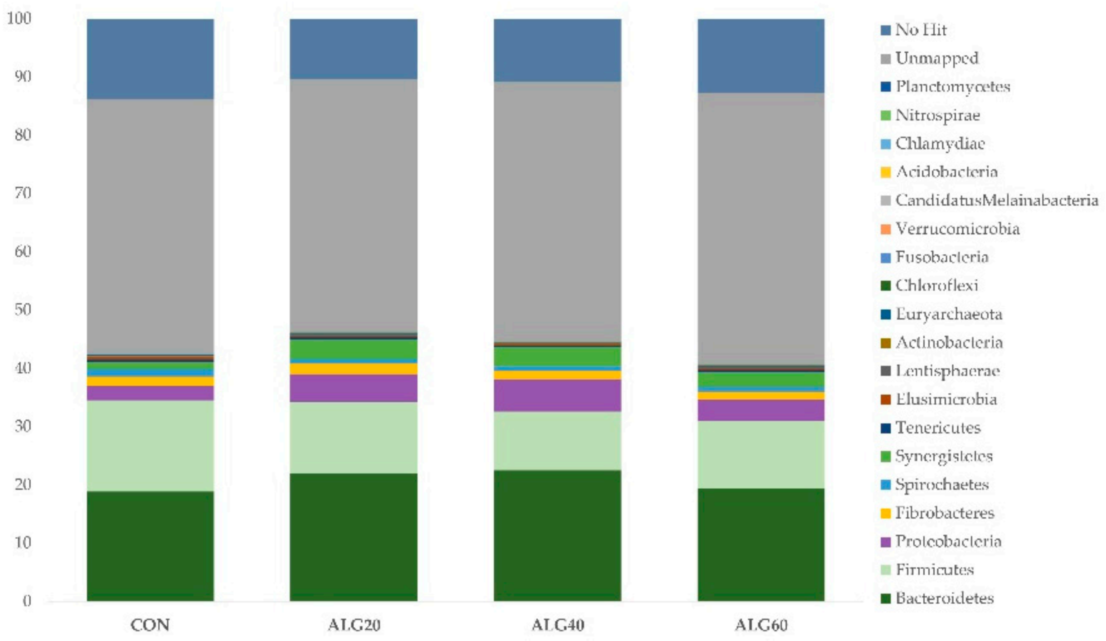

Figure S3. Relative abundance of identified phyla, unidentified clusters, and no-hits sequences in four dietary treatments (CON, ALG20, ALG40, and ALG60) within the two sampling times (20th and 40th) illustrated in cumulative bar graph.

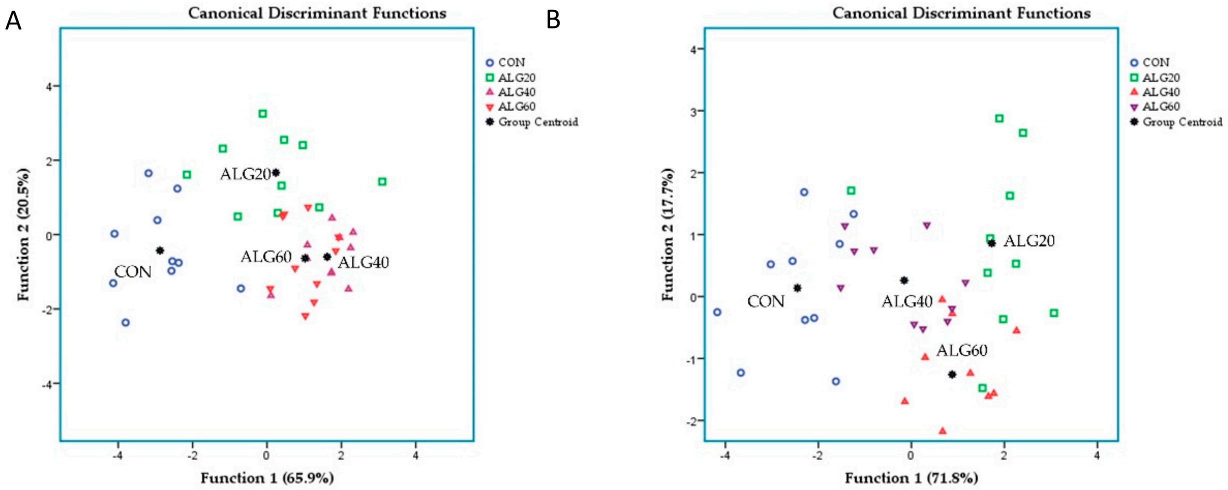

Figure S4. Discriminant plots separating (**A**) the four dietary treatments (CON; blue ○, ALG20; green □, ALG40; purple △, and ALG60; red ▽) according to pooled data of two sampling times (20th and 40th experimental day) that entered independents together on the abundances explained A) the fourteen identified phyla and B) the thirty-four identified families in goats' rumen. In the (A) plot the proportions of the samples that were correctly classified were 80 %, while wilks' λ was observed at 0.067 for Function 1 ($p < 0.001$) and 0.291 for Function 2 ($p = 0.075$). In the (**B**) plot the proportions of the samples that were correctly classified were 82.5 %, while wilks' λ was observed at 0.115 for Function 1 ($p = 0.013$) and 0.429 for Function 2 ($p = 0.496$). CON = control concentrate without microalgae (*Schizochytrium* spp); ALG20 = CON with 20 g/Kg *Schizochytrium* spp.; ALG40 = CON with 40 g/Kg *Schizochytrium* spp.; ALG6 0= CON with 60 g/Kg *Schizochytrium* spp.
